# A vector-encoded bispecific killer engager to harness virus-activated NK cells as anti-tumor effectors

**DOI:** 10.1038/s41419-023-05624-3

**Published:** 2023-02-10

**Authors:** Alessia Floerchinger, Jessica E. Klein, Maximiliane S. C. Finkbeiner, Theresa E. Schäfer, Gwendolin Fuchs, Johannes Doerner, Hubert Zirngibl, Maximilian Ackermann, Hans M. Kvasnicka, Kerry A. Chester, Dirk Jäger, Claudia R. Ball, Guy Ungerechts, Christine E. Engeland

**Affiliations:** 1 Clinical Cooperation Unit Virotherapy, German Cancer Research Center (DKFZ), National Center for Tumor Diseases (NCT), Heidelberg, Germany; 2 Center for Biomedical Education and Research (ZBAF), Institute of Virology and Microbiology, Faculty of Health, School of Medicine, Witten/Herdecke University, Witten, Germany; 3 Medical Faculty, Heidelberg University, Heidelberg, Germany; 4 Department of Surgery, Helios University Hospital Wuppertal, Wuppertal, Germany; 5 Institute of Pathology and Molecular Pathology, Helios University Clinic Wuppertal, Witten/Herdecke University, Witten, Germany; 6 UCL Cancer Institute, University College London, London, UK; 7 Department of Medical Oncology, University Hospital Heidelberg, Heidelberg, Germany; 8 Department of Translational Medical Oncology, National Center for Tumor Diseases (NCT/UCC), Dresden, Germany: German Cancer Research Center (DKFZ), Heidelberg, Germany, Faculty of Medicine and University Hospital Carl Gustav Carus, Technische Universität Dresden, Dresden, Germany; Helmholtz-Zentrum Dresden - Rossendorf (HZDR), Dresden, Germany; 9 German Cancer Consortium (DKTK), Dresden, Germany; 10 Translational Medical Oncology, Faculty of Medicine and University Hospital Carl Gustav Carus, Technische Universität Dresden, Dresden, Germany; 11 Technische Universität Dresden, Faculty of Biology, Technische Universität Dresden, Dresden, Germany; 12 Present Address: Francis Crick Institute, London, UK

**Keywords:** Cancer, Applied immunology

## Abstract

Treatment with oncolytic measles vaccines (MV) elicits activation of immune cells, including natural killer (NK) cells. However, we found that MV-activated NK cells show only modest direct cytotoxic activity against tumor cells. To specifically direct NK cells towards tumor cells, we developed oncolytic measles vaccines encoding bispecific killer engagers (MV-BiKE) targeting CD16A on NK cells and carcinoembryonic antigen (CEA) as a model tumor antigen. MV-BiKE are only slightly attenuated compared to parental MV and mediate secretion of functional BiKE from infected tumor cells. We tested MV-BiKE activity in cocultures of colorectal or pancreatic cancer cells with primary human NK cells. MV-BiKE mediate expression of effector cytokines, degranulation and specific anti-tumor cytotoxicity by NK cells. Experiments with patient-derived pancreatic cancer cultures indicate that efficacy of MV-BiKE may vary between individual tumors with differential virus permissiveness. Remarkably, we confirmed MV-BiKE activity in primaryhuman colorectal carcinoma specimens with autochthonous tumor and NK cells.This study provides proof-of-concept for MV-BiKE as a novel immunovirotherapy to harness virus-activated NK cells as anti-tumor effectors.

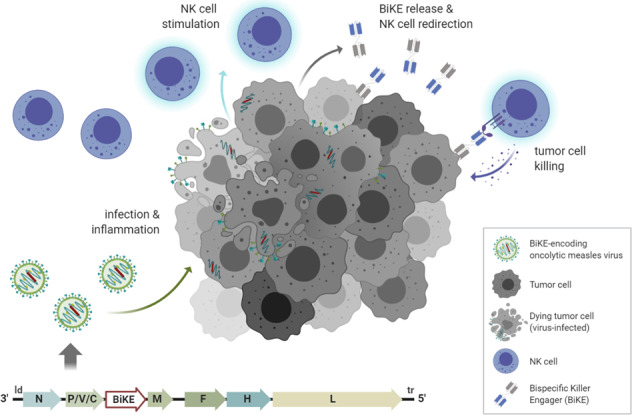

## Introduction

Oncolytic viruses (OVs) preferentially infect and replicate in malignant cells, inducing immunogenic tumor cell lysis. Based on this oncotropism, OVs are emerging anticancer agents and promising vectors for tumor-targeted delivery of therapeutic transgenes [1]. Attenuated, replication-competent measles vaccine virus (MV) provides a safe and versatile platform for oncolytic virotherapy [2, 3]. Tumor-selective cell entry and MV replication are associated with overexpression of the complement regulatory protein CD46 and a defective cellular interferon response in malignantly transformed cells [4]. Apart from direct cytotoxicity by lytic replication, immune modulation is crucial for effective virotherapy. MV has been shown to elicit innate and adaptive anti-tumor immunity by promoting several steps in the “cancer immunity cycle”, i.e., antigen presentation, immune infiltration, and T cell activation [5]. The in situ tumor vaccination effect [6] can be enhanced with MV engineered to encode immunomodulators [7, 8], seeking to enable combination treatment even if systemic administration of respective therapeutics may be limited by severe toxicity. However, despite remarkable progress in the field of immunovirotherapy, little is known about the role and therapeutic potential of natural killer (NK) cells in MV therapy [5].

NK cells are innate lymphoid cells regulated by activating and inhibitory receptors. Their main effector functions are killing of virally infected or malignant cells and pro-inflammatory cytokine production [9]. While anti-tumor cytotoxicity is desirable in OV therapy, anti-viral activity may be detrimental to viral spread and potentially limit efficacy. Seemingly contradictory findings for different oncolytic viruses and tumor models demonstrate the ambivalent role of NK cells as a result of the delicate balance between anti-tumor and anti-viral effector functions [10, 11]. We hypothesized that NK cell redirection can enhance cytotoxicity against cancer cells as well as pro-inflammatory cytokine release in the context of oncolytic virotherapy and synergize with MV-dependent immune cell recruitment and stimulation.

To effectively redirect NK cells and exploit the cytolytic capacity for cancer immunotherapy, bispecific killer engagers (BiKEs) have been developed [12]. These artificial proteins trigger formation of an immunological synapse between effector and target cells via simultaneous binding to tumor-associated antigens (TAAs) on cancer cells and the CD16 Fc receptor on NK cells. Similar to IgG antibodies, BiKEs elicit antibody-dependent cell-mediated cytotoxicity (ADCC) by CD16 engagement and induce target cell death as well as cytokine production without the need for additional costimulation [13]. Compared to therapeutic antibodies, the CD16-targeting moiety of BiKEs can be optimized for CD16A selectivity, higher binding affinity irrespective of the CD16 allotype in patients [14], and recognition of alternative epitopes to circumvent competitive receptor binding with serum IgG. The smaller size may further elicit superior biodistribution and tumor penetration as well as faster blood clearance [15]. Initial efforts predominantly focused on NK cell engagement in hematologic diseases, wherefore clinical translation is most advanced for AFM13, a tetravalent bispecific anti-CD30/CD16A antibody. In phase I clinical trials, AFM13 was well tolerated both as monotherapy [16] and in combination with the PD-1 checkpoint inhibitor pembrolizumab [17]. Multiple NK cell engagers against solid tumors are in preclinical development with encouraging results [18, 19]. Moving forward, these strategies will, however, have to demonstrate safe and effective application for cancer patients in ongoing clinical trials (NCT04259450; NCT04143711; NCT04074746; [20]). We reasoned that tumor-directed delivery of NK cell engagers with an oncolytic vector will be beneficial, since intratumoral accumulation of the therapeutic may prevent potential side effects, whereas virus-mediated anti-tumor immune effects may potentiate efficacy. We hence employed measles vaccine strain virus to generate, for the first time to our knowledge, a BiKE-encoding oncolytic virus (MV-BiKE).

## Results

### Natural killer cell activity in oncolytic measles virotherapy and combination treatment with bispecific killer engagers

#### NK cell stimulation by virus-infected tumor cells

**Fig. 1:**
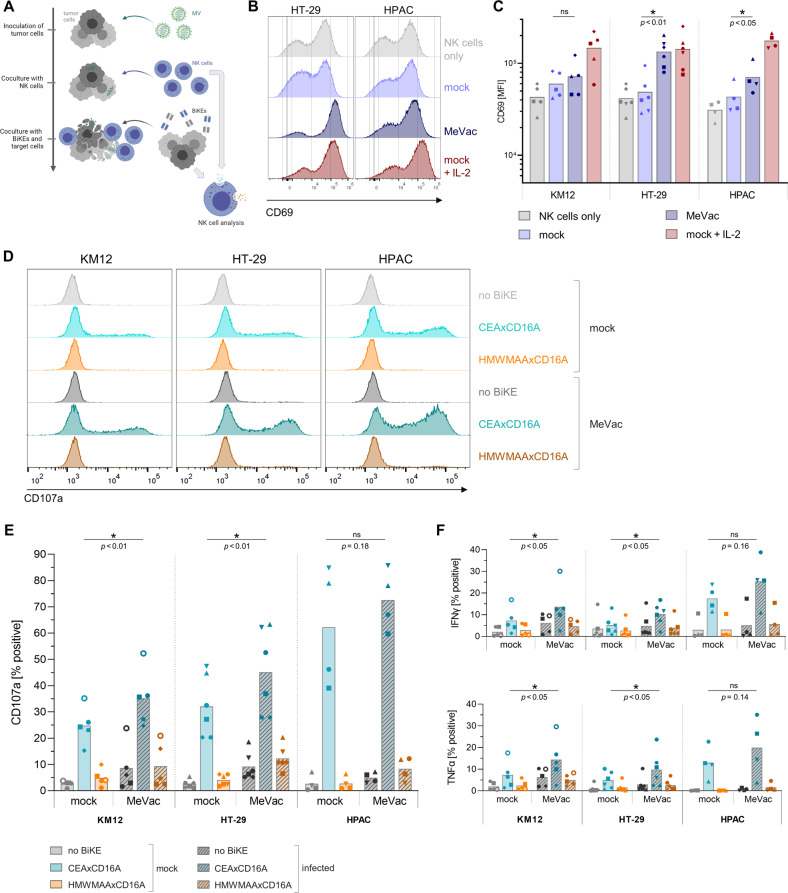
NK cell activation and effector functions in MV therapy combined with bispecific killer engagers. NK cell activation upon exposure to colorectal cancer (KM12, HT-29) or pancreatic ductal adenocarcinoma (HPAC) cells infected with recombinant measles vaccine strain virus (MV) was assessed in vitro. NK cell degranulation and cytokine expression were monitored in response to MV therapy with subsequent BiKE treatment. **A** Schematic outline of combination therapy experiments. Colorectal cancer (KM12, HT-29) or pancreatic ductal adenocarcinoma (HPAC) cells were inoculated with MV at a multiplicity of infection (MOI) of 1 or subjected to mock infection one day prior to coculture with NK cells isolated from healthy donor PBMCs. After 12 h co-incubation, i.e., 36 h post inoculation, expression of the early activation marker CD69 was analyzed by flow cytometry. NK cell only control and IL-2 stimulated positive control were included for comparison. In further experiments, BiKEs were purified from supernatants of infected Vero producer cells (vBiKEs). Non-infected target cells and vBiKEs were added to the coculture and incubated for 4 h. NK cell degranulation and cytokine expression were analyzed by flow cytometry. **B** Exemplary histograms depict expression levels of CD69 on live CD45^+^ CD56^+^ NK cells after coculture with infected cells compared to control conditions. **C** CD69 mean fluorescence intensity (MFI) is summarized for *n* ≥ 4 donors for each cell line with different symbols representing individual donors. **D** NK cell degranulation (CD107a) in response to MeVac and vBiKE monotherapy or combination therapy is shown in exemplary histograms for one donor. **E** Percentages of CD107a^+^ NK cells, **F** IFNγ^+^ and TNFα^+^ NK cells in different conditions are summarized for *n* ≥ 4 donors per cell line with different symbols representing individual donors. Statistical analysis was performed by paired t-test for each target cell line.

To assess natural killer cell activation by virotherapy, we cocultured NK cells with MV-infected colorectal cancer (CRC) and pancreatic adenocarcinoma (PDAC) cells and analyzed expression of the early activation marker CD69 (Fig. 1A(i)). Compared to NK cell only controls and mock treatment of tumor cells, CD69 expression on NK cells was increased after exposure to MV-infected tumor cells (Fig. 1B, C). The magnitude of increase varied between different tumor cell lines. Highest CD69 levels were observed in IL-2 stimulated positive controls. Analysis of MV entry receptors showed CD46 expression on NK cells (Fig. S1A). However, inoculation of NK cells with EGFP-encoding MV (MeVac ld-EGFP) did not result in considerable EGFP transgene expression, and low percentages of EGFP^+^ NK cells were detected in cocultures with MeVac ld-EGFP-infected tumor cells (Fig. S1B, C). In direct comparison, increased CD69 expression was only found after coculture with infected CRC cells, but not after inoculation of NK cells alone (Fig. S1D, E), indicating that exposure to infected cells rather than direct NK cell infection elicits NK cell stimulation. As NK cells seem to be activated by virus treatment of tumor cells, we hypothesized that combination treatment with bispecific killer engagers can direct NK cell cytotoxicity against malignant cells in the context of virotherapy.

#### Validation of BiKE functionality

BiKEs targeting CD16A on NK cells and carcinoembryonic antigen (CEA) as a model tumor antigen were encoded in recombinant MVs. Constructs targeting high molecular weight melanoma associated antigen (HMWMAA) as a control antigen not expressed in CRC and PDAC were generated accordingly. BiKE proteins were purified from supernatants of MV-BiKE-infected Vero producer cells (vBiKE, Fig. S2A) and tested for functionality. Specific binding to the targeted tumor antigen and CD16 on NK cells was confirmed by flow cytometry (Fig. S2B). Further, binding to CEA-positive CRC and PDAC cells was shown for the CEA-specific BiKE, but not the control construct (Fig. S2C). Cocultures of tumor and effector cells were prepared to monitor target cell killing and NK cell effector functions. Target cell death was increased in presence of the relevant vBiKE, demonstrating effective NK cell engagement and hence functionality of vBiKEs (Fig. S2D, E). In controls with either non-relevant BiKE protein or without treatment, baseline killing was observed compared to conditions without effector cells. NK cell degranulation (CD107a surface levels) as well as IFNγ and TNFα cytokine expression were enhanced upon specific BiKE treatment, whereas CD16 surface levels were decreased (Fig. S2F, G). Having validated vBiKE functionality, we proceeded to assess the combination of MV therapy and BiKE treatment.

#### NK cell effector functions in combination treatment of MV with BiKEs

To address NK cell responses to BiKE engagement after exposure to virus-infected cells, we cocultured NK cells with MV-infected tumor cells and subsequently added vBiKEs as well as non-infected tumor cells (Fig. 1A(ii), Fig. 1D—F). MV treatment alone resulted in a marginal increase of NK cell degranulation and cytokine expression levels compared to mock conditions. NK cell effector functions were strongly elevated upon combination therapy with the tumor-binding CEAxCD16A vBiKE, but not for the non-binding HMWMAAxCD16A vBiKE. NK cell degranulation as well as IFNγ and TNFα cytokine expression levels upon MV plus vBiKE combination treatment exceeded levels observed after respective vBiKE monotherapy. We concluded that combining virotherapy and BiKEs is beneficial.

Recombinant MV generated in the laboratory by standard procedures, i.e., clarification of infected cell lysate, may contain both viral gene products and other cellular components that stimulate NK cells. In contrast, clinical virotherapy protocols employ highly purified virus products. MV-NIS is such a measles-derived virotherapeutic that is currently investigated in clinical trials (NCT03171493; NCT02962167; NCT02068794; NCT02364713; NCT02700230) [21]. Therefore, we compared NK cell responses to lab-grade Schwarz (MeVac) or Edmonston (NSe) vaccine strain stocks and highly purified MV-NIS derived from the Edmonston strain.

We extended stimulation experiments presented in Fig. 1. NK cell coculture with infected tumor cells for 12 h resulted in slightly increased CD69 expression for MeVac compared to mock infection, as shown before, which was however not observed for MV-NIS (Fig. S3A). Subsequent coculture with non-infected target cells and vBiKEs elicited high NK cell degranulation for CEAxCD16A vBiKEs, as described above. Compared to vBiKE monotherapy (mock), the percentage of CD107a^+^ NK cells was increased upon MeVac, but not MV-NIS infection after 12 h coculture (Fig. S3B). We speculated that different infection kinetics of these virus strains might result in varying NK cell responses and hence monitored CD69 levels at three designated time points post inoculation.

Indeed, after longer coculture time spans, increased CD69 expression compared to mock controls was observed for HT-29 cocultures treated with MV-NIS (S3C). In addition, combination therapy with target-specific vBiKE starting after the 36 h coculture period elicited elevated cytokine expression and IFNγ accumulation was marginally increased upon MV-NIS compared to mock infection (Fig. S3D, E). Similarly, for KM12 target cells, vBiKE treatment after 36 h coculture triggered stronger NK cell degranulation in the MV-NIS plus CEAxCD16A condition compared to mock infection, even though no increase in CD69 expression was observed on NK cells. In direct comparison with MV-NIS and MeVac, these findings also extend to lab-grade NSe (Fig. S3F, G).

In conclusion, NK cells strongly respond to BiKE therapy after exposure to infected tumor cells, and this finding extends to clinically relevant MV-NIS, albeit kinetics of stimulation differ between MV variants.

### Therapeutic efficacy of MV-BiKE in vitro

**Fig. 2:**
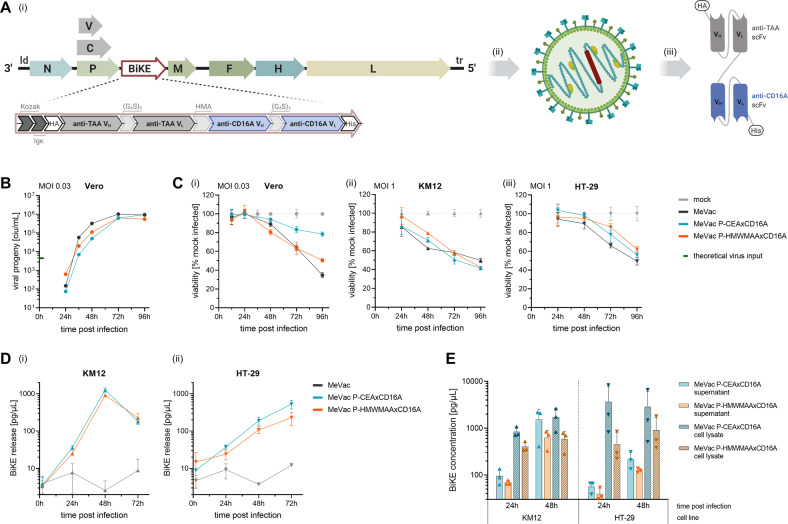
Oncolytic measles virus encoding BiKEs. **A** Schematic of recombinant MV-BiKE. (i) The BiKE transgene is encoded downstream of the *P* open reading frame within the MV genome. Hemagglutinin (HA) and hexa histidine (His_6_) tags enable purification and detection. Kozak and Igκ leader sequences mediate efficient translation and secretion. (G_4_S)_3_, glycine-serine peptide linker; HMA, human muscle aldolase linker; scFv, single chain variable fragment with variable heavy (V_H_) and variable light (V_L_) chain; TAA, tumor-associated antigen. (ii) Transgene-encoding virus can be rescued via a reverse genetics system and triggers (iii) BiKE secretion upon infection. BiKEs included in this study target either human carcinoembryonic antigen (CEA) as model tumor antigen or human high molecular weight melanoma-associated antigen (HMWMAA) as a non-relevant control. **B** Replication kinetics of MV-BiKE. Vero producer cells were inoculated with MeVac P-BiKE or unmodified MeVac at MOI 0.03. Viral progeny at designated time points were determined via serial dilution titration assay to obtain a multistep growth curve. **C** Direct cytotoxic effect of MV-BiKE. Cells were infected with respective viruses at MOI 0.03 for Vero (i) or at MOI 1 for colorectal cancer cell lines KM12 (ii) and HT-29 (iii). Cell viability was determined via XTT assay and normalized to mock-infected control. **D**, **E** BiKE expression kinetics on infected colorectal cancer cells. KM12 and HT-29 cells were inoculated with MeVac P-BiKE or MeVac at MOI 1. Supernatants and cell lysates were collected at indicated time points. BiKE concentrations were determined via ELISA using anti-His_6_ and anti-HA antibodies and purified vBiKEs as standard. **D** Kinetics of BiKE concentrations in culture supernatants after infection with MV-BiKE. **E** Concentrations of cell-associated BiKE in tumor cell lysates and BiKE release into culture supernatants. In **C**–**E** mean values of technical triplicates are shown. Error bars indicate SD.

As opposed to oncolytic virotherapy combined with systemic BiKE treatment, co-delivery of BiKE proteins as therapeutic payload of OVs can enable local transgene expression in the tumor microenvironment. Having shown that MeVac and vBiKE administered separately elicit enhanced NK cell effector functions, we further characterized MV encoding BiKEs to realize this concept (Fig. 2A). Specifically, we generated live-attenuated MV Schwarz vaccine strain vectors harboring a BiKE open reading frame (MeVac P-BiKE).

#### Characterization of BiKE-encoding MV for oncolytic combination therapy

MV-BiKE replication kinetics were compared to unmodified MV via titration of viral progeny after infection of Vero producer cells (Fig. 2B). The direct cytopathic effect of MV-BiKE on Vero cells and human colorectal cancer cell lines was assessed by cell viability assay (Fig. 2C). BiKE-encoding viruses were mildly attenuated compared to unmodified control. KM12 cells were found to be more susceptible to MV-mediated cytolysis compared to HT-29 cells. Kinetics of BiKE release into the culture supernatant consequently differed between both cell lines as determined by ELISA, with de novo BiKE production confirmed for both. For KM12, the highest BiKE concentrations of approximately 1 ng/µL were measured 48 h post infection with MV-BiKE viruses, whereas the highest concentrations for HT-29 were measured 72 h post infection (Fig. 2D). Concentrations of cell-associated BiKE protein in cell lysates showed different kinetics compared to BiKE released into the supernatant, with higher concentrations in lysates already detected 24 h post infection (Fig. 2E).

#### MV-BiKE-mediated NK cell cytotoxicity

**Fig. 3:**
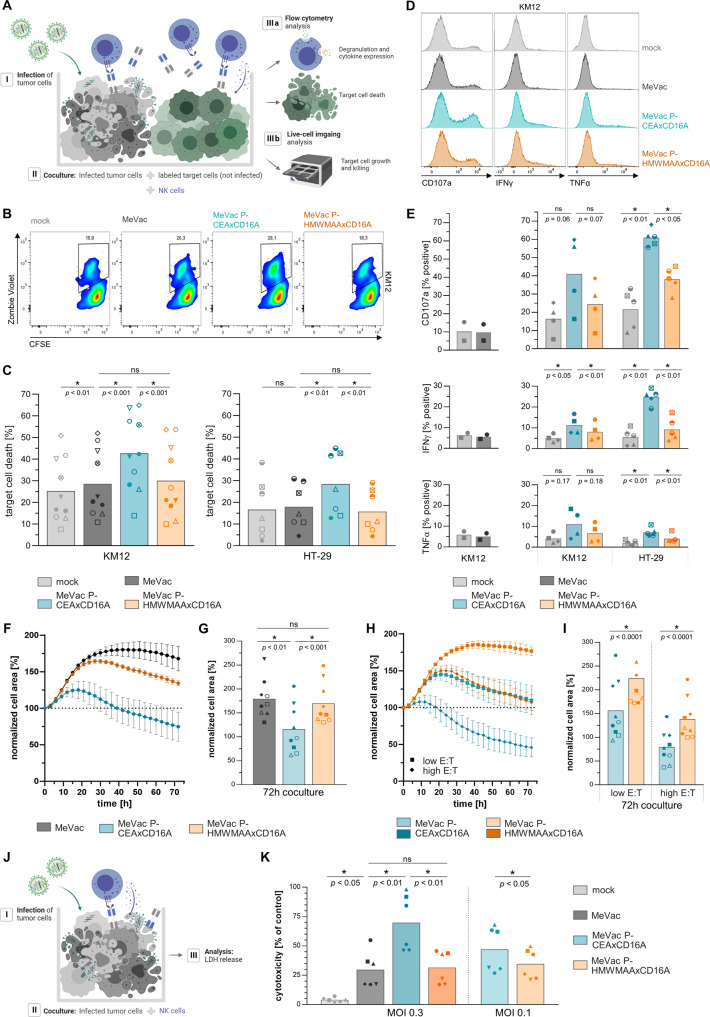
Efficacy of MV-BiKE immunovirotherapy against infected and bystander colorectal cancer cells. **A** Schematic outline of in vitro coculture experiments. MV-BiKE infected colorectal cancer cells were cocultured with NK cells and non-infected bystander tumor cells labeled with CFSE or tagRFP. NK cell degranulation, cytokine expression and killing of CSFE^+^ target cells were analyzed by flow cytometry. The tagRFP^+^ tumor cell area was monitored for 72 h using live-cell imaging. Recombinant MV encoding the tumor-targeting CEAxCD16A BiKE are compared to mock-infected control, MV without transgene and MV encoding an HMWMAAxCD16A control BiKE with irrelevant specificity. **B**–**E** Flow cytometry analysis of MV-BiKE efficacy. **B** Exemplary pseudocolor plots for KM12 depict CFSE^+^ target cell viability based on Zombie Violet labeling of dead cells. **C** Mean bystander target cell death per treatment is shown for *n* = 10 donors (KM12) and *n* = 7 donors (HT-29) with individual donors represented by different symbols. **D** NK cell degranulation and cytokine expression in response to MV-BiKE therapy. Exemplary histograms for KM12 target cells illustrate CD107a surface levels and intracellular cytokine accumulation. **E** Mean percentages of CD107a^+^, IFNγ^+^ and TNFα^+^ NK cells, respectively, are summarized for *n* = 4 donors (KM12) and *n* = 5 donors (HT-29) with individual donors represented by symbols as in **C**. Mock controls compared to MeVac are shown separately for two donors (left panels). **F**–**I** Live-cell imaging analysis of MV-BiKE efficacy against HT-29. Dotted line indicates 100%. **F** Exemplary time course plots show the kinetics of bystander tumor cell area during coculture. Mean and SD for three donors in one out of three independent experiments is shown (compare Fig. S4). **G** Normalized cell area after 72 h coculture is summarized for *n* = 9 donors. **H** Time course plots as in (**F**) illustrate efficacy of MV-BiKE treatment at higher and lower E:T ratios (shown in darker and lighter color, respectively), with quantification for *n* = 9 donors in three independent experiments in (**I**). **J** Schematic depicting the coculture of MV-BiKE infected HT-29 cells with NK cells for analysis of LDH release into culture supernatants. **K** Cytotoxicity in LDH release assays is summarized for *n* = 6 donors. Statistical analysis was performed by paired *t*-test for comparison of two groups (**I**) and one-way ANOVA with Šidák’s multiple comparisons test for more than two paired groups (**C**, **E**, **G**, **K**).

The potential of BiKE co-delivery as a therapeutic MV transgene was assessed in coculture assays of human healthy donor NK cells and MV-BiKE-infected tumor cells. To monitor specific cytotoxicity towards bystander cells as the critical target population, killing of non-infected tumor cells was quantified (Fig. 3A). Donor-dependent variability of baseline killing was observed in mock-infected controls, and NK cell cytotoxicity was enhanced upon MeVac treatment alone. Infection with MeVac encoding tumor-specific CEAxCD16A BiKE significantly increased the mean KM12 target cell death compared to MeVac and MeVac P-HMWMAAxCD16A controls, respectively (Fig. 3B, C). Similar trends were observed for HT-29 target cells. In controls without effector cells, bystander cells were not affected by direct cytotoxicity of different MV-BiKE constructs (Fig. S5F). In line with increased tumor cell death, highest NK cell degranulation and cytokine expression were found in cocultures treated with MeVac P-CEAxCD16A (Fig. 3D, E). Real-time cell analysis provided further evidence of MV-BiKE efficacy (Fig. 3F–I, Fig. S4): Cell area as a measure of tumor cell abundance decreased in cocultures with tumor cells treated with MeVac P-CEAxCD16A compared to virus-infected controls. Cytotoxicity assays confirmed increased tumor cell killing upon infection with MeVac encoding the CEA-targeting BiKE compared to controls (Fig. 3J, K). In summary, MV-BiKE effectively mediated NK cell activation and specific cytotoxicity against cancer cells.

As outlined above, virus preparations obtained from clarified crude cell lysate of infected Vero cells contain BiKE protein and other cellular components. We therefore purified MV-BiKE via ultracentrifugation to reduce BiKE concentrations in the inoculum (Fig. S5A). We compared the efficacy of purified viruses to conventional lab-grade stocks. Reduced concentration of protein per cell infectious unit and in particular lower BiKE content were confirmed via western blot and BiKE binding assay (Fig. S5B, C). BiKE concentrations in supernatants and lysates from infected tumor cells were found to be similar for purified and lab-grade viruses (Fig. S5D). In coculture assays, purified MV-BiKE efficiently triggered specific target cell killing, NK cell degranulation and cytokine accumulation, comparable to conventional MV-BiKE stocks (Fig. S5E–H). This indicates that de novo BiKE production from virus-infected cells occurs and mediates MV-BiKE efficacy.

### Characterization of NK cells in response to MV-BiKE

**Fig. 4:**
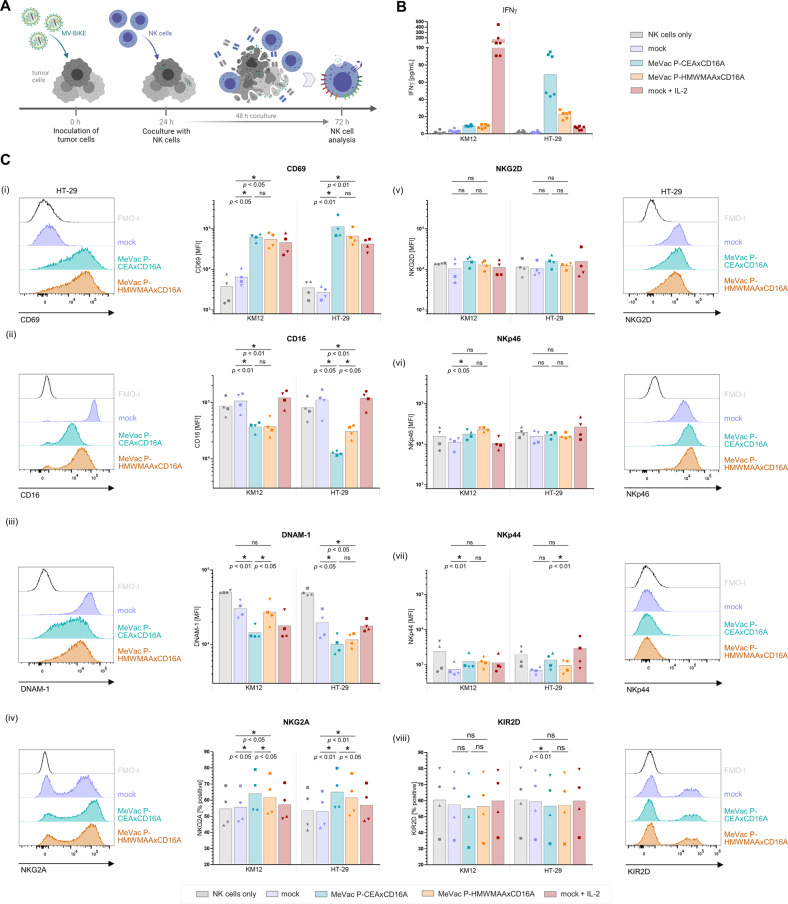
NK cell characterization in response to MV or MV-BiKE therapy. NK cell surface markers and IFNγ release were analyzed after coculture with infected colorectal cancer cells. MV-BiKE infections were compared to NK cell only controls, mock or IL-2 stimulation. **A** Schematic outline of the experiment. **B** IFNγ concentrations in coculture supernatants were quantified by ELISA. Results are shown for KM12 and HT-29 tumor cells with technical triplicates for *n* = 2 donors, represented by different symbols. **C** Characterization of NK cell surface markers by flow cytometry. Exemplary histograms from one donor are depicted including Fluorescence Minus One + Isotype (FMO-I) controls for each marker. Mean fluorescence intensity (MFI) or the percentage of cells expressing the respective marker are summarized for *n* = 4 donors. Different symbols represent individual donors. For CD16, the mean of three technical replicates is shown per donor. For additional data on MV alone or combined with purified vBiKEs, refer to Fig. S6. Statistical analysis was performed by one-way ANOVA with Šidák’s multiple comparisons test.

Given the functionality and efficacy of MV-BiKE, we characterized NK cells in response to measles virotherapy and MV-BiKE treatment in more detail. In addition, MV-BiKE was compared to the combination of MV and vBiKE administered separately. Cytokine release, CD69, and activating as well as inhibitory NK cell receptors were analyzed after 48 h exposure to infected cells (Fig. 4A). MeVac P-CEAxCD16A infection elicited increased IFNγ levels compared to MeVac and MeVac P-HMWMAAxCD16A controls in HT-29 cocultures (Fig. 4B), in line with enhanced NK cell cytokine expression in MV-BiKE efficacy experiments (compare Fig. 3E). Further, MV infection of HT-29 combined with tumor-binding CEAxCD16A vBiKE treatment resulted in higher IFNγ concentrations compared to the non-binding control (Fig. S6A). For KM12, IFNγ concentrations were close to the detection limit in all conditions apart from the IL-2 stimulated control. The CD69 activation marker was increased on NK cells in all MV-infected conditions (Fig. 4C(i), S6B(i)), indicating MV-mediated NK cell stimulation as described above (compare Fig. 1 and S1). CD16 Fc receptor levels were decreased both for MV-BiKE and MV + vBiKE treatment, which was most pronounced for conditions with tumor-specific, CEA-targeting BiKEs (Fig. 4C(ii), S6B(ii)). Surface expression of the adhesion molecule and activating receptor DNAM-1 was decreased upon MV infection, with strongest reductions found in CEAxCD16A conditions (Fig. 4C(iii), S6B(iii)). The NKG2A inhibitory NK cell receptor appeared to be slightly increased in coculture with MV-infected tumor cells compared to mock. CEAxCD16A-encoding viruses or combination therapy with CEAxCD16A vBiKEs were also found to slightly increase NKG2A compared to respective HMWMAA-targeting controls (Fig. 4C(iv), S6B(iv)). No consistent effects were observed for the activating receptors NKG2D, NKp46, and NKp44, or the killer immunoglobulin-like receptor (KIR) 2D subtype including activating and inhibitory isoforms (Fig. 4C(v–viii), S6B(v–viii)). In summary, MeVac treatment led to increased expression of the NK cell activation marker CD69 on NK cells and BiKE treatment led to a decrease in CD16 levels. Additional effects on activating and inhibitory NK cell receptors were either subtle or cell line-dependent, indicating that NK cell phenotype upon MV-BiKE treatment may differ between individuals.

### NK cell engagement with scFv-Fc constructs as alternative MV transgene

MVs provide a flexible platform to deliver therapeutic transgenes, so that different payloads can be tested to optimize NK cell stimulation and tumor-directed cytotoxicity. In addition to MV-BiKE, we generated analogous MVs encoding scFv-Fc proteins (MV-scFv-Fc, Fig. S7A). These engagers are composed of an IgG1Fc portion fused to the tumor-targeting scFv, resulting in conventional Fc receptor binding on NK cells and other immune cell subsets such as macrophages. Binding of virus-encoded scFv-Fcs to CEA-expressing tumor and CD16^+^ NK cells was confirmed by flow cytometry, with high signals detected on NK cells compared to MV-BiKE, presumably resulting from the dimeric structure of functional scFv-Fc (Fig. S7B). Further, binding to in vitro differentiated macrophages was confirmed (data not shown).

In coculture assays, MeVac P-CEAxIgG1Fc infection led to increased bystander target cell death compared to controls with non-relevant scFv-Fc transgene and mock infection (Fig. S7C, D). Compared to respective MV-BiKE, no consistent difference in therapeutic efficacy was observed across the two cell lines tested. Infection with relevant MV-scFv-Fc further resulted in increased NK cell degranulation and cytokine expression, particularly on HT-29 target cells (Fig. S7E, F). Of note, enhanced NK cell degranulation and IFNγ accumulation upon infection with MV encoding non-relevant HMWMAA-scFv-Fc compared to unmodified MV did not correlate with increased HT-29 target cell death. Characterization of NK cell surface markers in response to MV-scFv-Fc therapy revealed high levels of CD69 in all MV-infected conditions compared to mock, with strongest NK cell stimulation in the MeVac P-CEAxIgG1Fc condition. CD16 surface levels were strongly reduced upon exposure to MV-scFv-Fc-infected tumor cells, which was most pronounced for the tumor-binding, CEA-targeting transgene. In contrast to BiKEs, binding of scFv-Fc proteins to NK cells was found to interfere with subsequent CD16 detection by flow cytometry to a limited extent. The percentages of NKG2A^+^ NK cells were slightly increased and DNAM-1 levels decreased upon MeVac P-CEAxIgG1Fc infection compared to control (Fig. S7G). Changes in NK cell surface marker expression hence showed similar trends as observed for BiKE-encoding viruses (compare Fig. 4C). Taken together, MV-scFv-Fcs are functional, and in vitro efficacy was confirmed to be similar to MV-BiKE in terms of NK cell engagement.

### MV-BiKE efficacy against patient-derived pancreatic cancer cultures

**Fig. 5:**
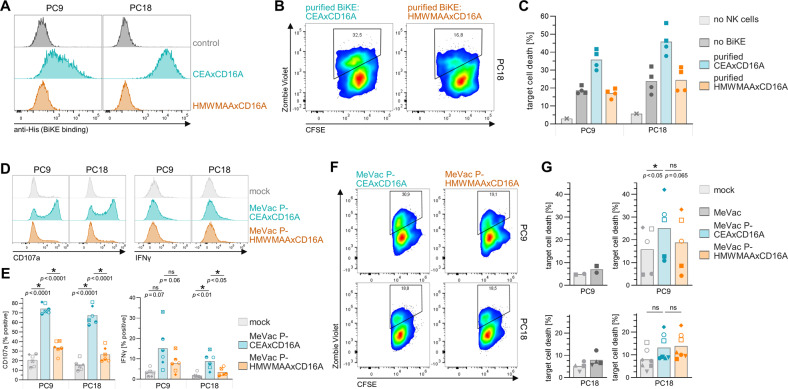
MV-BiKE efficacy against patient-derived pancreatic cancer cultures. **A** BiKE binding to pancreatic cancer cultures. BiKEs were purified from supernatants of Vero producer cells infected with respective viruses (vBiKEs). Cancer cells were incubated with CEAxCD16A vBiKEs or the HMWMAAxCD16A control. Binding was assessed via flow cytometry based on BiKE labeling with anti-His_6_ antibodies. Histograms for PC9 and PC18 are depicted. **B**, **C** NK cell cytotoxicity upon vBiKE treatment. Allogenic, IL-2 pre-stimulated NK cells were co-incubated with CFSE-labeled PC9 or PC18 cultures (E:T = 5:1) in presence of vBiKEs (5 ng/µL) for 12 h with subsequent analysis of target cell death via flow cytometry. **B** Pseudocolor plots exemplarily illustrate gating and CFSE^+^ tumor cell viability for PC18 based on Zombie Violet staining of dead cells. **C** Target cell death for *n* = 2 donors with technical duplicates per donor is shown. **D**–**G** NK cell activity upon infection of patient-derived cultures with MV-BiKE. Infected cancer cells were cocultured with CFSE-labeled, non-infected target cells and allogenic, IL-2 pre-stimulated NK cells for 12 h (compare Fig. 3A). NK cell degranulation, cytokine expression, and target cell killing were analyzed by flow cytometry. **D** NK cell degranulation (CD107a) and intracellular IFNγ accumulation in response to MV-BiKE therapy are depicted in exemplary histograms. **E** Mean percentages of CD107a^+^ and IFNγ^+^ NK cells are shown for *n* = 6 donors with different symbols representing individual donors. **F** Pseudocolor plots display the viability of non-infected CFSE^+^ target cells in coculture with respective MV-BiKE infected cancer cells and NK cells. **G** Mean percentages of target cell death in designated cocultures are summarized for n = 5 donors (PC9, top) and *n* = 7 donors (PC18, bottom) with donors represented by symbols as in (**E**). Statistical analysis was performed by one-way ANOVA with Šidák’s multiple comparisons test.

Having demonstrated MV-BiKE efficacy in cell lines, patient-derived pancreatic cancer cultures were used to study our therapeutic approach in a model system which has been shown to closely reflect patient tumor histology and cellular heterogeneity [22, 23]. Among seven cultures screened, we identified two cultures, PC9 and PC18, with strong CEA-specific BiKE binding (Fig. 5A). Co-incubation with allogenic healthy donor NK cells resulted in considerable baseline killing, which was enhanced in presence of specific vBiKEs (Fig. 5B, C). The cultures showed intermediate to low sensitivity towards MV infection, whereas PC9 was found to be more sensitive than PC18 (Fig. S8). In the next step, efficacy of MV-BiKE infection to elicit BiKE-mediated NK cell cytotoxicity towards bystander cells was investigated (compare Fig. 3A). For both cultures, increased NK cell degranulation and IFNγ expression were detected for MeVac P-CEAxCD16A compared to controls (Fig. 5D, E). However, all infected conditions resulted in similarly enhanced tumor cell killing compared to mock control for PC18, whereas increased target cell death and hence therapeutic efficacy of MeVac P-CEAxCD16A against bystander cells was observed for PC9 (Fig. 5F, G). Hence, MV-BiKE treatment was effective against PC9, the more permissive culture.

### MV-BiKE activity in primary colorectal cancer

**Fig. 6:**
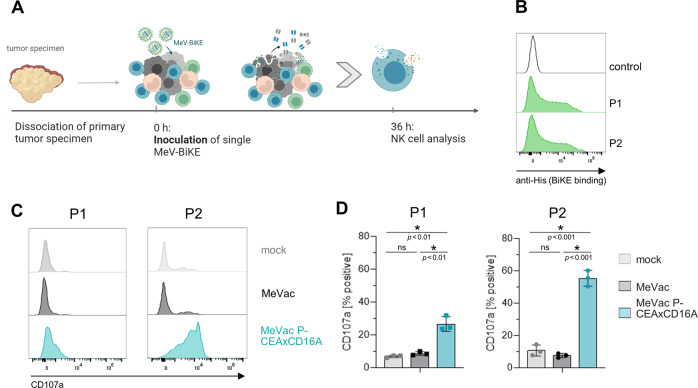
MV-BiKE activity in primary colorectal cancer. NK cell degranulation was analyzed after infection of single cell suspensions of patient-derived colorectal carcinoma specimens. MV-BiKE infections were compared to MV infection and mock controls. **A** Schematic outline of ex vivo experiments. **B** BiKE binding to single cell suspensions of patient-derived colorectal carcinoma. Cell suspensions were incubated with CEAxCD16A vBiKEs. Binding was assessed via flow cytometry (gated on all viable cells) based on BiKE labeling with anti-His_6_ antibodies. Histograms for patient 1 (P1) and patient 2 (P2) are depicted. **C**, **D** NK cell granulation upon MV-BiKE treatment. Colorectal carcinoma single cell suspensions were infected and NK cell degranulation was analyzed by flow cytometry. **C** NK cell degranulation (CD107a) in response to MV-BiKE therapy is depicted in exemplary histograms. **D** Mean percentages of CD107a^+^ NK cells are shown for *n* = 3 technical replicates of *n* = 2 patients (P1, left; P2, right) with different symbols representing individual patients.

To test MV-BiKE activity in an autochthonous setting, we employed an ex vivo culture model of single cell suspensions of primary colorectal carcinoma specimens (Fig. 6A). Two independent specimens were obtained, which both showed binding of CEAxCD16A vBiKEs (Fig. 6B). In both specimens, treatment with MeVac led to an increase in CD69 fluorescence intensity on NK cells compared to mock treatment (Fig. S9A, B). A significant increase in the percentage of CD107a^+^ NK cells was detected upon treatment with MeVac P-CEAxCD16A compared to mock and MeVac treatment (Fig. 6C, D). Interestingly, viable NK cell counts were increased after MeVac and especially MeVac P-CEAxCD16A treatment (Fig. S9D).

These results demonstrate that MV and MV-BiKE treatment activate NK cells in an ex vivo culture model that genuinely represents the cellular composition of human primary colorectal carcinomas.

## Discussion

Oncolytic viruses trigger immunogenic cell death and local inflammation, which promote the recruitment of immune cells, including NK cells, to the TME. Stimulated NK cells can eradicate infected or malignant cells and potentially contribute to anti-tumor immunity in oncolytic virotherapy. However, the role of NK cells in oncolytic MV therapy remains to be unraveled in more detail, and may be two-sided: As opposed to anti-tumor effects, potent anti-viral capacity of NK cells can be detrimental for OV efficacy by limiting viral spread and mediating premature viral clearance [10]. We reasoned that redirection of NK cells via BiKEs in the context of OV therapy will strengthen cytotoxicity against malignant cells, including non-infected bystander cells, and tilt the balance towards anti-tumor activity. Further, combination of BiKEs with OV-induced tumor debulking and immune stimulation might help to overcome challenges anticipated for clinical application of BiKE therapy in solid tumors, such as limited immune infiltration and hyporesponsiveness in the immunosuppressive TME.

We report stimulation of human healthy donor NK cells exposed to MV-infected colorectal or pancreatic cancer cells, as indicated by increased CD69 expression compared to mock-infected controls. Release of NK cell effector proteins including perforin, granzymes, and cytokines has previously been reported for cocultures of activated and expanded NK cells with MV-infected sarcoma cells [24]. Importantly, the present study shows that NK cell effector functions are enhanced upon combination of MV infection with purified vBiKE. Notably, the product MV-NIS, which is administered in clinical trials of measles virotherapy, elicited effects comparable to laboratory-grade virus preparations. Overall, these data indicate that the combination of MV plus BiKE is beneficial, encouraging further development of this approach.

Toxicity of systemically applied immunomodulators including BiKEs may limit tolerability. Therefore, we sought to exploit the oncolytic vector for tumor-targeted expression of the therapeutic BiKE transgene. With this study, we provide an in vitro proof-of-concept for BiKE-encoding measles vaccine strain virus as a novel combination immunovirotherapy. Treatment of colorectal cancer cells with MV-BiKE elicited potent NK cell cytotoxicity against bystander cells, accompanied by NK cell degranulation and increased cytokine expression. Compared to separate administration of MV and BiKE, MV-BiKE is expected to yield localized, intratumoral BiKE accumulation and reduced systemic exposure. Indeed, we confirmed BiKE production from infected tumor cells in vitro. However, biodistribution of OV-encoded engagers in patients can only be inferred from early clinical trials with MV-NIS, in which the encoded human sodium iodide symporter enables imaging of radioiodine uptake to monitor viral replication. After intraperitoneal or intravenous administration of MV-NIS in ovarian cancer or multiple myeloma patients, respectively, positive SPECT/CT scans indicated viral gene expression at the tumor site in a subset of patients [25, 26]. Data from clinical trials with MV encoding secretable CEA as marker protein (NCT00390299, NCT00408590) may inform on pharmacokinetics of transgene products after intratumoral and locoregional OV administration [27]. Moving forward, analysis of pertinent clinical samples will be crucial to evaluate viral (trans)gene expression and stimulation of NK cells in treated patients.

Apart from total intratumoral BiKE concentrations, the timing of BiKE exposure with regard to viral replication kinetics and immune responses can be decisive for optimal efficacy. Characterization of MV-BiKE confirmed viral replication and tumor cell cytolysis with only mild attenuation compared to MV without transgene. BiKE release into the culture supernatant was detected prior to the onset of considerable cytotoxicity, indicating BiKE secretion from infected cells. Timing of MV-induced immune recruitment, NK cell stimulation, and BiKE-mediated cytotoxicity could be modulated by distinct dosing and scheduling regimens or advanced oncolytic vectors (e.g., with inducible transgene expression) as well as combination therapies.

Determinants of NK cell chemoattraction and stimulation in MV therapy remain to be deciphered. Understanding the underlying signaling mechanisms would allow optimization of MV-BiKE therapy. Direct NK cell receptor binding to viral proteins or upregulation of known NK cell ligands on cancer cells upon infection and oncolysis have not been described to date for MV, in contrast to several other OVs [28]. Besides upregulation of the early activation marker CD69, we report decreased levels of DNAM-1 and increased NKG2A expression on NK cells in coculture with MV-infected tumor cells, which is most pronounced in combination treatments with either tumor-specific MV-BiKE, or MV plus BiKE. DNAM-1 binds to nectin/nectin-like family molecules CD112 and CD155, and modulation of these activating ligands is known to be involved in viral infection and immune evasion of multiple viruses [29], albeit not described for MV infection in particular. Conversely, DNAM-1 upregulation has previously been reported on NK cells cocultured with tumor cells and BiKE [30]. The NKG2A receptor mediates lymphocyte inhibition via HLA-E binding and is considered an immune checkpoint for both NK and CD8^+^ T cells. In line with our finding, an increase in NKG2A surface expression on NK cells has previously been shown upon monotherapy with either BiKE [30] or MV [24]. NKG2A blockade with the monoclonal antibody monalizumab is under clinical investigation as cancer immunotherapy [31]. Thus, combination approaches with NKG2A checkpoint inhibition are conceivable. Likewise, MV has been combined with immune checkpoint blockade [32–34], and disinhibiting lymphocyte function, e.g. by NKG2A-, KIR-, TIGIT- or PD-1-specific antibodies [35], may augment MV-BiKE efficacy. However, MV-BiKE therapy exploits CD16A engagement to provide a strong stimulus and trigger cytotoxicity independent of additional costimulation. Reduced CD16 levels on NK cells upon exposure to tumor antigen-specific BiKEs, in line with previous reports of CD16 loss in BiKE and therapeutic antibody approaches [30], could arguably limit NK cell responses. Consequently, BiKE therapy has successfully been combined with metalloprotease ADAM17 inhibition to prevent CD16 shedding and enhance NK cell effector functions in vitro [36]. Intriguingly however, NK cell reactivity and CD16 expression have been shown to be recovered via cultivation with IL-2 and IL-15, with enhanced cytolytic capacity after CD16A engagement [30]. Consequently, modulation of the NK cell phenotype by MV is not only important for immediate BiKE-mediated cytotoxicity, but may have implications for long-term NK cell functionality in MV and MV-BiKE therapy.

Since the overall immune contexture is remodeled during MV infection, other immune cell subsets will additionally affect MV-BiKE therapy. Pleiotropic effects of MV oncolysis have been described to support activation of both innate and adaptive anti-tumor immunity [5], but data regarding the impact on NK cells in the TME remain scarce. The interaction between NK cells and other immune cells is presumably bidirectional, with NK cell stimulation also enhancing DC functions, for instance [37]. Preliminary data indicate that NK cells upregulate CD69 and CD107a upon co-culture with MV-exposed DCs [38]. In vitro experiments with oncolytic reovirus showed that DCs loaded with infected tumor cells secreted NK chemotactic factors and induced CD69 upregulation and IFNγ expression in NK cells, which in turn promoted DC maturation [39]. Fueling NK cell cytotoxicity and cytokine release in the context of virotherapy via BiKE engagement might therefore support anti-tumor effects beyond NK cell-dependent killing, specifically OV-induced tumor vaccination effects.

This notion is supported by induction of adaptive anti-tumor immunity in response to alternative NK cell engagement approaches in the context of oncolytic therapy, as described in previous studies. Firstly, treatment with an adenovirus encoding an adapter protein to redirect antiviral antibodies towards tumor cells delayed tumor growth and elicited NK cell-dependent CD8^+^ T cell responses in adenovirus-immunized mice [40]. Secondly, herpes simplex virus (HSV)-derived OVs have been engineered for expression of chimeric molecules to redirect immunoglobulins (Igs) towards tumor cells, which enhanced NK cell infiltration and reduced tumor burden, accompanied by stimulation of neoantigen-specific adaptive immunity in the CT26 murine colon cancer model [41]. Compared to BiKEs, antibody redirection approaches might benefit from a dampened antiviral response, but can be limited by competitive Fc receptor binding to serum Igs not captured by engager proteins. Aiming to combine OV therapy with direct engagement of infiltrating immune cells, a recent study described co-delivery of bispecific engagers with HSV via an amplicon vector, which elicited activation and cytotoxicity of both NK and CD8^+^ T cells via NKG2D binding [42].

In earlier studies, virotherapy was employed for tumor-targeted immune cell redirection with a focus on T cells. To broaden the clinical applicability of bispecific T cell engagers against solid tumors in particular, multiple OVs have successfully been engineered to encode such immunomodulators [43], including oncolytic measles vaccine strain virus [44]. Treatment with MV encoding T cell engagers was shown to enhance T cell infiltration, induce anti-tumor immunity in syngeneic mouse models, and exert anti-tumor efficacy in patient-derived xenograft mouse models of solid tumors. With the development of MV-BiKE for NK cell engagement and tumor-directed cytotoxicity in the context of MV-mediated immune stimulation, we expand the available treatment modalities.

Besides cell-cell interactions with stimulated NK cells, additional immune cells in the TME can directly respond to virus-encoded immunomodulators. Direct comparison of MVs encoding either BiKE or scFv-Fc proteins confirmed effective NK cell degranulation and target cell killing for both constructs. BiKEs, scFv-Fcs, and other formats for NK cell engagement not only differ with respect to CD16 allotype affinity, tissue penetration due to size, and competition for receptor binding with serum immunoglobulins [15], but also regarding binding to immune cells with Fc receptors, apart from NK cells. For therapeutic application, effects on additional immune cell populations need to be considered, although not addressed experimentally in this study. For instance, crosslinking of tumor cells and macrophages can induce antibody-dependent cellular phagocytosis (ADCP). CD16A-specific immune engagers have been shown to bind to in vitro differentiated human macrophages and mediate ADCP [], such that macrophage functions may have an impact on MV-BiKE therapy. Engineered oncolytic herpes simplex virus encoding CD47 targeting antibodies have been used to disrupt the CD47-SIRPα signaling axis and enhance both macrophage-mediated ADCP and NK cell-mediated ADCC, harnessing the overexpression of the CD47 “don’t eat me” signal on many tumor cells [45, 46]. Similar to NK cells, tumor-associated macrophages play a dichotomous role in oncolytic virotherapy, either supporting or hindering oncolysis and inflammation [11, 47]. For MV therapy, one in vitro study reported reduced viability of MV-infected cancer cells in coculture with human monocyte-derived macrophages, accompanied by macrophage repolarization towards an anti-tumor M1 phenotype [48]. Enhanced phagocytosis via Fc receptor engagement can be desirable to drive antigen presentation and adaptive immunity, but further studies are needed to characterize the macrophage phenotype in MV therapy and resulting implications for the MV-BiKE approach.

Alternative NK cell engager formats have been investigated preclinically, including tri- or tetraspecific killer engagers that integrate stimulatory cytokines or simultaneously target multiple NK cell receptors [49]. An anti-CD33 trispecific killer engager containing an IL-15 moiety has entered clinical testing against hematological malignancies (NCT03214666). MV provides a flexible platform to compare virus-encoded engagers, such that these multivalent immunomodulators could be incorporated. However, limitations in transgene capacity and virus attenuation resulting in reduced direct cytotoxicity and intratumoral replication should be taken into account. One key rationale for the integration of cytokine moieties into engager molecules is the NK cell-restricted stimulation upon binding, as opposed to systemic administration with potential toxicity. Since engineered OVs allow targeted delivery of therapeutic transgenes to the TME, combination treatment with cytokine-encoding MV could equally be considered, and simultaneous T cell activation might boost overall efficacy. Accordingly, MV encoding an IL-15 superagonist mediated increased infiltration of both NK and T cells in syngeneic mouse models. IL-12 encoding vectors promoted robust immune activation, with superior efficacy in direct comparison [8, 50]. Ultimately, rational design of treatment schedules and synergistic therapies will require further mechanistic insights into the intricate crosstalk between different immune cell populations in MV and BiKE therapy.

In this regard, adequate models are mandatory to gain relevant insights. Although widely employed in preclinical immunotherapy studies, syngeneic mouse models have several shortcomings with respect to the MV-BiKE approach: MV is a strictly human-adapted virus [51], therefore murine models do not reflect the pharmacokinetics and -dynamics of MV-derived virotherapeutics. Further, human NK cells exhibit functional differences compared to murine counterparts [52, 53]. Thus, we chose to employ primary human NK cells in this study and tested efficacy of MV-BiKE in ex vivo cocultures with human colorectal and pancreatic cancer cell lines as well as patient-derived pancreatic cancer cultures. Remarkably, we confirmed MV-BiKE activity in primary human colorectal carcinoma specimens with autochthonous tumor and NK cells. As BiKE target, we used CEA as an exemplary tumor antigen relevant for gastrointestinal tumors. We detected de novo expression of MV-encoded BiKEs in human cancer cells, albeit secreted BiKE levels were considerably lower in the less MV-permissive cell line. While CD107a and IFNγ expression were elevated in MV-BiKE cocultures of patient-derived pancreatic cancer cultures, increased BiKE-mediated bystander killing was only observed in cocultures of the more MV-sensitive culture. Although limited by sample size and the non-autologous setting inherent to our protocols, these observations may suggest that MV-BiKE efficacy requires a certain degree of tumor cell permissiveness to achieve meaningful BiKE-mediated effects. Aside from tumor cell-intrinsic factors, the tumor microenvironment and individual patient’s immune status will affect therapeutic activity of this approach. Predictive biomarkers for measles virotherapy and NK cell-mediated immunotherapy remain to be identified. The flexibility of the MV platform would allow to incorporate alternative BiKE target antigens as well as additional immunomodulators to account for individual tumor-specific factors.

The mechanisms of MV-mediated NK cell activation remain to be resolved and the clinical indications for this approach are yet to be identified. Nevertheless, this study demonstrates that virus-stimulated NK cells can be harnessed efficiently by vector-encoded BiKE to augment anti-tumor cytotoxicity.

## Materials and Methods

### MV-BiKE vectors

BiKEs were constructed based on an affinity-maturated, humanized anti-hCEA scFv (SM3E) [54] and the affinity-maturated anti-CD16A scFv (LSIV21) [14]. SM3E was modified for increased stability by introducing variable heavy (V_H_) –variable light (V_L_) disulphide-forming substitutions at Kabat positions R44C (V_H_) and G100C (V_L_) [55, 56]. The CD16A-targeting scFv is employed in engager formats tested in clinical trials [17] (NCT04259450), and Fc receptor binding is only marginally impaired by serum IgG []. Reverse translated nucleotide sequences were codon optimized for expression in *homo sapiens* by MV using the GENEius algorithm (Eurofins Genomics) [57]. V_H_V_L_ chains within each scFv were connected by artificial (Gly_4_Ser)_3_ peptide linkers. A linker derived from human muscle aldolase (HMA, PSGQAGAAASESLFVSNHAY, [58]) was inserted between the N-terminal anti-hCEA scFv and the C-terminal anti-CD16A scFv. Influenza virus hemagglutinin (HA) and hexa-histidine (His_6_) tags were fused to the N- and C-terminus of the BiKE sequence, respectively. The BiKE sequence was preceded by a 5’ Kozak sequence and an Igκ leader sequence (compare GenBank: AB050084.1). After synthesis of the BiKE cassette (Eurofins Genomics), the 1644 bp transgene fulfilling the *rule of six* [59] was inserted into a Schwarz vaccine strain-derived full length measles virus genome with an additional transcription unit downstream of the phosphoprotein open reading frame (MeVac P-ATU) [8]. To generate high molecular weight melanoma-associated antigen (HMWMAA)-targeting control constructs, the anti-hCEA scFv was replaced by an anti-hHMWMAA scFv (RAFT3) [60, 61]. Sequences were validated by Sanger sequencing (Eurofins Genomics).

The anti-hCEA scFv sequences employed were the following:

V_H_: QVKLEQSGAEVVKPGASVKLSCKASGFNIKDSYMHWLRQGPGQCLEWIGWIDPENGDTEYAPKFQGKATFTTDTSANTAYLGLSSLRPEDTAVYYCNEGTPTGPYYFDYWGQGTLVTVSS

V_L_: ENVLTQSPSSMSVSVGDRVTIACSASSSVPYMHWLQQKPGKSPKLLIYLTSNLASGVPSRFSGSGSGTDYSLTISSVQPEDAATYYCQQRSSYPLTFGCGTKLEIK

To obtain scFv-Fc constructs, the anti-CD16A scFv in respective BiKE sequences was replaced by human IgG1Fc (hinge-CH_2_-CH_3_) [32], eliminating the HMA linker.

### Cell culture

Cell lines, patient-derived cultures and NK cells were cultivated at 37 °C and 5% CO_2_ in humidified incubators. Cultures were routinely tested to exclude mycoplasma contamination.

#### Cell lines

Vero cells were obtained from the American Type Culture Collection (ATCC, CCL-81). KM12 and HT-29 cells were obtained from Christoph Plass, German Cancer Research Center Heidelberg. HPAC cells were obtained from Daniel Abate-Daga, H. Lee Moffitt Cancer Center and Research Institute, Tampa, FL.

Vero and KM12 cell lines were cultivated in Dulbecco’s modified Eagle’s medium (DMEM, Thermo Fisher Scientific and PAN Biotech) supplemented with 10% (v/v) fetal calf serum (FCS, PAN Biotech). HT-29 and HPAC cell lines were cultivated in Rosewell Park Memorial Institute (RPMI) 1640 medium (Thermo Fisher Scientific and PAN Biotech) with 10% (v/v) FCS.

#### Patient-derived pancreatic cancer cultures

Patient-derived pancreatic cancer cultures PC9 and PC18 were generated and cultivated as described previously [22]. Cultures were subjected to SNP typing and Multiplex Cell Contamination Testing (Multiplexion, Heidelberg, Germany). PC9 and PC18 cultures were grown in DMEM Advanced F12 medium supplemented with 0.6% (w/v) glucose, 2 mM L-glutamine, 2% B27 supplement (1×) (all Thermo Fisher Scientific), 12 μg/mL heparin and 5 mM HEPES buffer (both Sigma Aldrich), referred to as CSCN medium. Cytokines, i.e., 10 ng/mL rhFGF-basic, 20 ng/mL rhFGF-10, and 20 ng/mL rhNodal (all R&D Systems) were added to the cultures and renewed twice a week.

#### NK cells

Human peripheral blood mononuclear cells (PBMCs) were isolated by density gradient centrifugation of blood or buffy coats obtained from healthy donors. Buffy coats were provided by Deutsches Rotes Kreuz (DRK)-Blutspendedienst in Mannheim, Germany and DRK-Blutspendedienst West, Zentrum für Transfusionsmedizin Hagen (Hagen, Germany). Leukocyte filters containing PBMCs from whole blood were donated by BZD Gesellschaft für Transfusionsmedizin Duisburg mbH (Duisburg, Germany).

NK cells were purified via depletion of other immune cell subsets using the human NK Cell Isolation Kit (Miltenyi Biotec) according to the manufacturer’s instructions. NK cells were either directly used for experiments or cultured at 2 × 10^6^ cells/mL in RPMI medium supplemented with 10% human serum (Sigma Aldrich), 50 U/mL penicillin-streptomycin (Thermo Fisher Scientific), and 100 U/mL IL-2 (Proleukin, Clinigen) (pre-stimulated). Viability and purity of isolated NK cells was confirmed by flow cytometry (Zombie Violet^-^, CD45^+^, CD3^−^, CD19^−^, CD14^−^, and CD56^+^).

### Recombinant measles viruses

#### Virus rescue, propagation, and titration

Recombinant MV were generated via transfection of Vero cells with antigenomic cDNA constructs using the established RNA polymerase II-dependent reverse genetics system [62] as previously described [57]. Recombinant measles Schwarz vaccine strain MeVac, its derivative MeVac ld-EGFP [8], and the Edmonston B-derived measles vaccine strain NSe [44] have been described previously. For virus propagation, Vero cells were inoculated at a multiplicity of infection (MOI) of 0.03 in Opti-MEM (Thermo Fisher Scientific) and culture medium was added 2 h post infection. Cultures were incubated at 37 °C and 5% CO_2_, syncytia formation was monitored, and virus-containing cells were scraped 40–48 h post infection. Viruses were released by one freeze-thaw cycle, clarification was performed (2500 × g, 5 min) and aliquots of cell-free supernatant were stored at −80 °C. Infectious units in viral stocks were quantified by ten-fold serial dilution in octuplicates using 96-well plates with 1.5 × 10^4^ Vero cells per well. Individual syncytia were counted 48 h and 72 h post infection and titers calculated as cell infectious units (ciu)/mL. MV were passaged up to five times to obtain stocks for experimentation. Expression of functional BiKE transgenes from these stocks was confirmed by BiKE/scFv-Fc binding assays as described below.

Highly purified high titer MV-NIS was obtained from Imanis Life Sciences.

#### Viral infection, replication kinetics, and cytotoxicity

Cells were seeded one day prior to inoculation at designated MOIs. Virus suspensions were diluted in Opti-MEM, unless indicated otherwise, and medium only was applied for mock-infected controls. Inocula were replaced by respective culture media 2 h post infection.

Phase contrast and fluorescence microscopy images were taken with a Leica DMi8 microscope.

For multi-step growth curves, 1 × 10^5^ Vero cells in 12-well plates were inoculated at MOI 0.03 in triplicates, cells were scraped into the culture medium at designated time points and pooled per condition. After one freeze-thaw cycle, titers of clarified virus suspensions were determined by serial dilution titration in quadruplicates.

Cell viability was assessed by XTT (2,3-Bis-(2-methoxy 4-nitro-5-sulfophenyl)-2H-tetrazolium-5-carboxanilide) assay. Cells were inoculated at MOI 0.03 (Vero, 1 × 10^5^ cells, 12-well plate) or MOI 1 (KM12 and HT-29, 4.5 × 10^4^ cells, 48-well plate) in triplicates. At designated time points post infection, the Colorimetric Cell Viability Kit III (PromoCell) was used according to the manufacturer’s protocol. Viability was normalized to mock-infected controls at respective time points.

Cell lysis was assessed by lactate dehydrogenase (LDH) release assay. Patient-derived cultures (PC9 and PC18, 1 × 10^5^ cells, 12-well plate) were inoculated at MOI 0.03 and MOI 3 in triplicates, with virus suspensions diluted in CSCN medium. The inoculum was replaced with fresh CSCN medium supplemented with cytokines 2 h post infection. For 100% LDH release samples, mock-infected cells were scraped 24 h post inoculation, subjected to two freeze-thaw cycles for complete cell lysis and stored at −80 °C. Culture supernatants were collected at designated time points, centrifuged (380 × g, 5 min) and stored at −80 °C. LDH release into the supernatants was quantified using the CytoTox 96 Non-Radioactive Cytotoxicity Assay kit (Promega). Relative cytotoxicity was calculated by normalization to the 100% LDH release control of the respective culture.

#### Virus purification via ultracentrifugation

To reduce protein contamination in clarified crude cell lysates where indicated, recombinant viruses were purified via iodixanol fractionation ultracentrifugation with a protocol adapted from [63] by Gemma Pidelaserra-Martí. Thinwall UltraClear 5 mL tubes (Beckman Coulter) were disinfected with 70% (v/v) ethanol and 2 × 4 mL virus suspension per construct were loaded onto 500 μL 20% (w/v) OptiPrep (Axis Shield) in TE buffer (5 mM Tris, 1 mM EDTA, pH 7.4), underlayered with 500 μL 54% (w/v) OptiPrep in TE buffer. Tubes were subjected to ultracentrifugation in an SW50.1 rotor (Beckman Coulter) at 28000 rounds per minute (rpm) for 2 h at 8 °C without brake. The interphase containing purified virus was collected, diluted to a total volume of 3 mL in Opti-MEM and stored at −80 °C. The upper and lower fractions were kept for analysis.

### MV-encoded BiKE and scFv-Fc proteins

#### Purification of BiKEs from virus-infected cells

Virus-encoded BiKEs were purified from supernatants of infected Vero cells (vBiKEs). 6 × 10^6^ cells in 15 cm culture dishes were inoculated with respective MV-BiKEs at MOI 0.03 in OptiPRO SFM serum-free medium (Thermo Fisher Scientific), cultured at 37 °C, 5% CO_2_ and monitored for syncytia formation. Supernatants were collected, clarified (2500 × g, 5 min, 4 °C), and passed through a 0.22 µm pore size filter (Merck) before vBiKEs were purified by affinity exchange chromatography via the His_6_ tag. Ni-NTA Spin Columns or Superflow Columns (Qiagen) were used for small and large scale purification, respectively, following the manufacturer’s instructions. In brief, cell-free supernatants were applied to equilibrated columns allowing BiKE binding to the Ni-NTA resin. Columns were rinsed with wash buffers containing 10 mM and 20 mM imidazole. Immobilized BiKE proteins were recovered with elution buffer containing 500 mM imidazole. Eluates were washed with DPBS, concentrated in Amicon Ultra-15 Centrifugal Filter Units with Ultracel-10 membranes (Merck), and stored at −80 °C. Protein concentration in purified vBiKE aliquots was quantified by BCA assay using the Novagen BCA Protein Assay Kit (Merck) according to the manufacturer’s instructions.

#### Gel electrophoresis and western blot analysis

BiKE content in fractions obtained from the protein purification procedure or virus purification via ultracentrifugation was analyzed by gel electrophoresis with 4–20% TGX Stain Free Gels (Bio-Rad), stain-free imaging, and western blot. BiKEs were detected with anti-HA biotin (clone 3F10, 10 ng/µL, Merck), streptavidin-HRP (0.02 µg/mL, Dianova), and Clarity Western ECL Substrate (Bio-Rad). HRP-specific chemiluminescence was acquired using a ChemiDoc MP Imaging System (Bio-Rad).

#### Enzyme-linked immunosorbent assay (ELISA) for BiKE quantification

At designated time points post infection, supernatants from triplicate wells were collected, centrifuged at 400 × g for 5 min, and stored at −80 °C. Lysates were harvested by scraping into RIPA Buffer (Sigma Aldrich) supplemented with cOmplete protease inhibitor cocktail (Roche) and pooled per condition. Samples were snap-frozen in liquid nitrogen and stored at −80 °C. Thawed lysates were centrifuged (2500 × g, 5 min, 4 °C) and diluted with DPBS for analysis to compare lysates and supernatants.

In a sandwich ELISA approach, 96-well plates (Nunc MaxiSorp flat-bottom, Thermo Fisher Scientific) were coated with 100 ng anti-His antibody (clone 13/45/31, Dianova) in 100 µL DPBS per well. After blocking in DPBS with 5% FCS, samples were incubated for 2 h at room temperature. Purified vBiKEs were used as standard. BiKEs were detected with anti-HA biotin (clone 3F10, 100 ng/µL, Merck), streptavidin-HRP (0.1 µg/mL, Dianova), and 1-Step Ultra TMB ELISA Substrate (Thermo Fisher Scientific). Absorbance was measured using an Infinite 200 Pro microplate reader (Tecan) with i-control software (v 2.0.10.0).

#### BiKE/scFv-Fc binding assay

BiKE and scFv-Fc binding to NK cells or tumor cells was analyzed by flow cytometry. Proteins bound to tumor cells via the anti-TAA scFv were detected with antibodies targeting the C-terminal His_6_ tag. Antibodies targeting the N-terminal HA tag were applied to assess binding to NK cells via the anti-CD16A (BiKE) or IgG1Fc (scFv-Fc) moiety.

1 × 10^5^ cells were incubated with Zombie Violet fixable viability dye (1:5000, BioLegend) for 15 min in the dark. For NK cells, Human TruStain Fc block (1:40, Biolegend) was added. Cells were washed and incubated with purified vBiKEs (2 µL) or MV-BiKE and MV-scFv-Fc virus suspensions (20 µL) for 30 min on ice in the dark. NK cells were stained with anti-CD16-BV650 (clone 3G8, 1:100, BioLegend) in parallel. After washing, cells were labeled with anti-HA FITC (clone GG8-1F3.3.1, 1:20, Miltenyi Biotec) or anti-His_6_ FITC (clone 13/45/31, 1:20, Dianova). Samples were fixed with 1% PFA in DPBS and analyzed by flow cytometry. Unstained and single stained controls were used to control for unspecific antibody binding.

### Functional in vitro coculture assays

#### Target cell killing assays

##### Flow cytometry

Killing of tumor cells by IL-2 pre-stimulated NK cells was assessed using a flow cytometry-based cytotoxicity assay (adapted from [64]). In principle, non-infected target cells were labeled with 5 µM Celltrace CFSE (Thermo Fisher Scientific) according to the manufacturer’s instructions and killing was quantified after co-incubation with NK cells and either vBiKEs or MV-BiKE-infected tumor cells as previously described [65].

To analyze vBiKE efficacy, 4 × 10^4^ CFSE-labeled cells were cocultured with 2 × 10^5^ NK cells and indicated vBiKE concentrations for 12 h. Cocultures were performed in TC-treated U-bottom plates with 200 µL RPMI supplemented with 10% FCS.

To analyze MV-BiKE efficacy, 4 × 10^3^ target cells in TC-treated U-bottom plates were infected at MOI 1 (KM12) or MOI 5 (HT-29, patient-derived cultures), and medium only for mock controls. Inocula were replaced by RPMI, 10% FCS and cultures were incubated for 48 h (KM12) or 72 h (HT-29, patient-derived cultures). To set up cocultures, 2 × 10^4^ CFSE-labeled, non-infected target cells (bystander cells) and 2 × 10^5^ NK cells were added to the infected cells and incubated for 12 h. Conditions were performed in duplicates and pooled for analysis.

For quantification of target cell death, supernatants were collected and pooled with adherent cells from respective conditions after detachment with 0.05% Trypsin EDTA (Thermo Fisher Scientific). Cells were stained with Zombie Violet fixable viability dye (1:2000, BioLegend). After washing and fixation, samples were analyzed by flow cytometry. Unstained and single stained tumor cell controls were included for gating and at least 5000 CFSE^+^ events were recorded. Samples were gated on CFSE^+^ non-infected tumor cells and the percentage of Zombie Violet^+^ dead cells was determined.

NK cell degranulation and cytokine expression were analyzed in parallel to target cell killing. Cocultures were prepared as outlined above, omitting the CFSE-labeling of non-infected target cells. Positive controls were stimulated with 50 nM PMA (Cayman Chemical) and 1 µM ionomycin (Cayman Chemical). To detect degranulation events, anti-CD107a antibodies were added directly to the cultures. Protein transport inhibitors GolgiStop (1 μL/mL, BD Biosciences) and GolgiPlug (1 μL/1.5 mL, BD Biosciences) containing monensin and brefeldin A, respectively, were added 1 h after setting up the cocultures. After 12 h incubation, cells were harvested. Surface markers (CD45, CD16, CD56) and intracellular cytokines (IFNγ, TNFα) were stained for analysis by flow cytometry.

##### Real-time cell analysis

Real-time cell analysis of bystander tumor cells in MV-BiKE infected cocultures was performed using an Incucyte SX5 instrument. For imaging purposes, HT-29 target cells were lentivirally transduced to stably express tagRFP using pRSI9-U6-(sh)-UbiC-TagRFP-2A-Puro (Addgene #28289) and tumor cell area was quantified during co-incubation with NK cells and MV-BiKE-infected tumor cells. To analyze MV-BiKE efficacy, 5 × 10^3^ target cells were infected at MOI 1 or medium only for mock controls in TC-treated flat-bottom plates. Inocula were replaced by RPMI, 10% FCS and cultures were incubated for 72 h. To set up cocultures, 1 × 10^4^ tagRFP expressing, non-infected target cells and 5 × 10^4^ (low E:T), 1 × 10^5^ or 2 × 10^5^ (high E:T) NK cells were added to the infected cells and imaged for 72 h in intervals of 3 h in the IncuCyte SX5 instrument starting 1 h post coculture setup. Four images per well from three technical replicates were taken. Acquisition time for the orange channel (excitation: 546–568 nm, emission: 576–637 nm; tagRGP imaging) was 400 ms. The analysis module “Basic analyzer” was used to quantify non-infected bystander cells by measuring tagRFP^+^ fluorescent objects. An area filter was applied to exclude objects below 50 µm^2^. Orange channel background noise was subtracted with the Top-Hat method of background non-uniformity correction with a radius of 100 µm and a threshold of 0.75 orange corrected units. Fluorescence signal was quantified applying a mask using edge split. These analysis settings were applied to three independent experiments. For each well, the cell area was normalized to the first imaging time point (= 100%).

##### LDH release assay

For quantification of HT-29 target cell death using LDH release assay, 1 × 10^4^ target cells were inoculated with MV at MOI 0.3 and 0.1, or medium only for mock controls in TC-treated 96-well U-bottom plates. Inocula were replaced by RPMI, 10% FCS and cultures were incubated for 72 h. To set up cocultures, 1 × 10^5^ NK cells were added to the infected cells. Culture supernatants were collected after 48 h and LDH release was measured using the Cytotox96® Non-Radioactive Cytotoxicity Assay (Promega) according to manufacturer’s instructions. Absorbance was measured using an Infinite 200 Pro microplate reader (Tecan) with i-control software (v 2.0.10.0) at 492 nm. Three technical replicates were performed for each condition and blank values as determined in medium only wells were subtracted from all measurements. Cytotoxicity was calculated using the following formula:

$$\% \,{{{\mathrm{Cytotoxicity}}}} = \frac{{{{{\mathrm{well}}}}\,{{{\mathrm{of}}}}\,{{{\mathrm{interest}}}} - {{{\mathrm{NK}}}}\,{{{\mathrm{cells}}}}\,{{{\mathrm{spontaneous}}}} - {{{\mathrm{HT}}}} {\hbox{-}} {{{\mathrm{29}}}}\,{{{\mathrm{spontaneous}}}}}}{{{{{\mathrm{HT}}}} {\hbox{-}} {{{\mathrm{29}}}}\,{{{\mathrm{maximum}}}} - {{{\mathrm{HT}}}} {\hbox{-}} {{{\mathrm{29}}}}\,{{{\mathrm{spontaneous}}}}}} \times 100$$

#### Analysis of NK cell infection with MV

Expression of MV entry receptors was analyzed on freshly isolated NK cells. Dead cells were excluded from the analysis based on Zombie Violet staining (1:5000, Biolegend). Cells were stained for CD46, CD150, or Nectin-4, and with respective isotype controls. As positive controls, HT-29 tumor cells and PBMCs were included.

NK cell infection and stimulation were analyzed either in coculture with infected tumor cells or upon direct inoculation of NK cells. In TC-treated 96-well U-bottom plates, 2 × 10^4^ tumor cells were infected with MV at MOI 1 or mock-infected in 100 µL Opti-MEM. MeVac ld-EGFP was used to monitor viral (trans)gene expression after infection, and NK cell stimulation was analyzed using MeVac without transgene. After 12 h, the inoculum was replaced by 150 µL RPMI, 10% FCS. Cocultures with 1 × 10^5^ NK cells were set up 24 h post infection. For inoculation of NK cells with MV at MOI 0.2 or MOI 1, virus suspension diluted in Opti-MEM was directly added to 1 × 10^5^ NK cells in TC-treated 96-well U-bottom plates. To replace the inoculum with fresh medium, plates were centrifuged at 800 × g for 2 min, supernatants were discarded, and 150 µL RPMI, 10% FCS was added. After incubation for 12 h to 60 h, either EGFP expression in live tumor (CD45^-^) and NK cells (CD45^+^) or CD69 levels on NK cells (CD45^+^, CD56^+^) were analyzed by flow cytometry, respectively.

#### NK cell analysis in MV plus vBiKE combination therapy

NK cell stimulation in the context of MV infection and NK cell effector functions in response to combination treatments with vBiKEs were addressed in coculture assays. 5 × 10^3^ tumor cells in TC-treated 96-well U-bottom plates were inoculated with MV at MOI 1 or mock-infected in 100 µL Opti-MEM. The inoculum was replaced by 150 µL RPMI, 10% FCS after 2 h. Cocultures with 2 × 10^5^ NK cells were set up 24 h post infection, including IL-2 (1000 U/mL) stimulated cocultures and NK cell only controls. After 12 h co-incubation, CD69 expression on NK cells was analyzed by flow cytometry.

BiKE combination treatment was started 36 h post infection, i.e., after 12 h coculture. To this end, 2 × 10^5^ non-infected target cells and vBiKEs (10 ng/µL) were added to the cocultures, and NK cell degranulation and cytokine expression were assessed. CD107a-specific antibodies, protein transport inhibitors and PMA/ionomycin for positive controls were applied as described above. After 4 h vBiKE treatment, i.e., 40 h post infection, cells were harvested. Surface markers (CD45, CD16, CD56) and intracellular cytokines (IFNγ, TNFα) were stained and NK cells were analyzed by flow cytometry.

For experiments with multiple analyses of CD69 expression at designated time points, cocultures were set up as described above with one replicate for each time point. NK cells (1 × 10^5^ cells) were added 24 h post infection and analyzed 36 h, 60 h, and 86 h post infection, i.e., after 12 h, 36 h, and 60 h in coculture, respectively. BiKE combination treatment was started 60 h post infection and analyzed 4 h later as described above.

#### NK cell characterization in MV-BiKE or MV-scFv-Fc therapy

NK cell surface receptor expression and IFNγ release were analyzed after exposure to MV, MV-BiKE, and MV-scFv-Fc-infected tumor cells, or to MV-infected cells with additional vBiKE treatment. For cocultures, 2 × 10^4^ tumor cells were inoculated at MOI 1, the inoculum was replaced by RPMI, 10% FCS 2 h post infection, and 1 × 10^5^ NK cells were added 24 h post infection. Purified vBiKEs (10 ng/µL) and IL-2 (1000 U/mL) were added to designated conditions directly after adding NK cells. Analysis was performed 72 h post infection, i.e., after 48 h coculture. IFNγ concentrations in coculture supernatants were quantified by ELISA using the IFN gamma Human Uncoated ELISA Kit (Thermo Fisher Scientific) according to the manufacturer’s instructions. NK cells were labeled with respective antibodies (panel 1: NKG2D, NKp46, DNAM-1; panel 2: NKG2A, KIR2D, NKp44; panel 3: CD69) and surface marker levels were analyzed by flow cytometry.

#### Ex vivo analyses with primary colorectal carcinoma samples

Fresh primary tumor material was obtained during colorectal cancer surgery and immediately assessed macroscopically by a specialist in pathology. Material was processed into pieces of approximately 1 cm^3^ and transferred to the laboratory in RPMI + 10% FCS + 1% penicillin/streptomycin on ice. Tumor pieces were minced with a scalpel in digestion buffer containing RPMI + 10% FCS and 200 U/mL collagenase type I. Minced tumor pieces were incubated for 30 min at 37 °C. Cell suspensions were then passed through a 100 µm cell strainer. Remaining cells were pelleted by centrifugation (5 min at 300 × g) and washed in PBS before MV inoculation and cultivation. For infection, triplicates of 2 × 10^6^ cells were inoculated with MV-BIKE, MV or OptiMEM (mock) for 2 h. After infection, cells were cultivated in RPMI supplemented with 10 % FCS and 1 % antibiotic/antimycotic solution for 36 h.

#### Flow cytometry analysis

To prepare NK cells for flow cytometry analysis, samples were incubated with Zombie Violet fixable viability dye (1:5000, Biolegend) and Human TruStain Fc block (1:40, Biolegend). NK cells were stained with antibodies specific for CD45, CD16 and CD56. For NK cell purity controls, stimulation and surface receptor analyses, further antibodies were added as indicated in respective sections. Samples were washed and fixed with 1% PFA in DPBS for analysis or fixed and permeabilized for subsequent intracellular cytokine staining using the Cytofix/Cytoperm Fixation/Permeabilization Kit (BD Biosciences). At least 10000 live CD45^+^ CD56^+^ NK cell events were recorded.

Surface marker antibodies: CD45-BV510, CD45-APC/Cy7 (clone HI30, 1:200); CD16-BV650, CD16-FITC, CD16-PerCP/Cy5.5 (clone 3G8, 1:100); CD56-APC/Cy7, CD56-PE (clone 5.1H11, 1:200), CD56-FITC (clone 5.1H11, 1:50); CD69-BV785, CD69-APC (clone FN50, 1:100); NKG2D-APC (clone 1D11, 1:200); NKp44-APC (clone P44-9, 1:100); NKp46-PE (clone 9E2, 1:200); DNAM-1-PE/Cy7 (clone 11A8, 1:200), CD107a-BV785, CD107a-Alexa Fluor 488 (clone H4A3, 1:200, added during coculture); CD46-APC (clone TRA-2-10, 1:100) (BioLegend); CD3-V450 (clone UCHT-1, 1:100); CD19-V450 (clone HIB19, 1:100); CD14-V450 (clone MφP9, 1:100); CD150-PE (clone A12, 1:20) (BD Biosciences); NKG2A-PE-Vio770 (clone REA110, 1:100); KIR2D-PE (clone NKVFS1, 1:100) (Miltenyi Biotec); Nectin-4-Alexa Fluor 488 (clone 337516, 1:50) (R&D Systems).

Intracellular cytokine antibodies: IFNγ-Alexa Fluor 647 (clone 4 S.B3, 1:100); TNFα-PE, TNFα-BV510 (clone Mab11, 1:100) (BioLegend); IFNγ-Alexa Fluor 647 (clone B27, 1:25) (BD Biosciences).

Compensation was calculated based on single stained compensation beads (OneComp eBeads Compensation Beads, Thermo Fisher Scientific; BD CompBeads, BD Biosciences) and gating was based on respective Fluorescence Minus One plus Isotype (FMO-I) controls. NK cell purity was confirmed after isolation, and NK cells were gated as single cell, live CD45^+^ CD56^+^ events for analysis of coculture assays.

Flow cytometry was performed on a FACS Fortessa instrument with FACS Diva software (v 8.0.1, BD Bioscience) or a CytoFLEX flow cytometer with CytExpert software (v 2.3, Beckman Coulter).

### Statistical analysis and data visualization

Flow cytometry data was analyzed and visualized using FlowJo (v 10.8.0, Tree Star Inc.). Statistical analyses were performed using GraphPad Prism software (v 9.1.2, GraphPad Software). Data was analyzed by paired t-test for two groups or one-way ANOVA for more than two groups comparing selected pairs of means as indicated, and *p* values were adjusted for multiple comparisons by Šidák’s test. For flow cytometry-derived MFI values, statistics was performed on log-transformed data. *P*-values < 0.05 were considered statistically significant.

Schematics in Figs. 1A, 2A, 3A, 3J, 4A, S5A, S7A, and the graphical abstract were created with BioRender.com.

## Supplementary information

Supplementary Figures

Reproducibility checklist

Authorship confirmations

## Data Availability

The data generated in this study are available upon request from the corresponding author.
