# Supplementary material for: A vector-encoded bispecific killer engager to harness virus-activated NK cells as anti-tumor effectors
Source: Cell Death Dis. 2023 Feb 10;14(2):104. doi: 10.1038/s41419-023-05624-3 (PMC9918448; doi:10.1038/s41419-023-05624-3)
Supplement: Supplementary file 1 — Supplementary Figures [file 41419_2023_5624_MOESM1_ESM.pdf]

Supplementary Figure 1

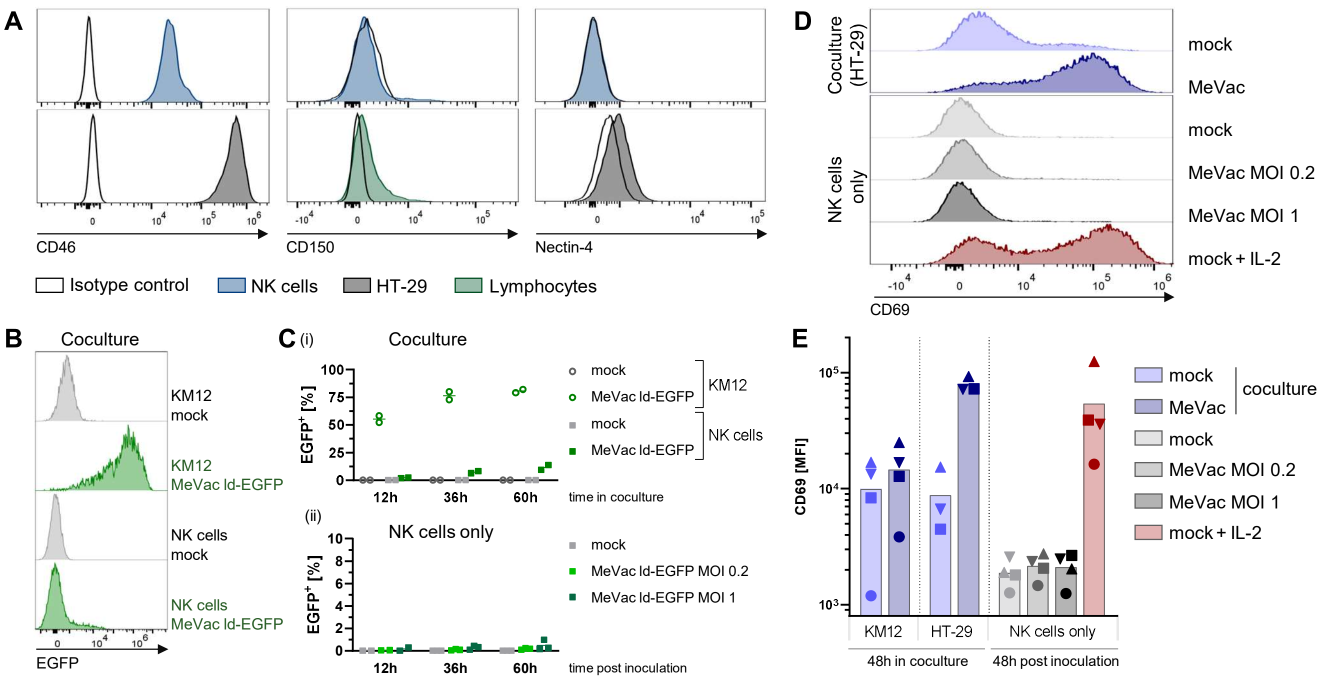

NK cell infection and stimulation in MV therapy

(A) Expression of MV entry receptors CD46, CD150 (SLAM) and Nectin-4 on NK cells purified from healthy donor PBMCs was analyzed by flow cytometry. Lymphocytes, gated from PBMCs based on FSC/SSC signals, and HT-29 tumor cells are depicted as controls. Representative histograms for n = 2 donors are shown. (B) – (E) KM12 tumor cells were inoculated with MeVac ld-EGFP or MeVac at MOI 1. After 24 h, NK cells were either added to the infected cells, or inoculated directly with MV at MOI 0.2 and MOI 1, respectively. EGFP expression in live (Zombie Violet$^-$) KM12 and NK cells as well as CD69 levels on NK cells were analyzed by flow cytometry at designated time points. (B) Exemplary histograms after 60 h coculture and (C) percentages of EGFP$^+$ tumor and NK cells in comparison to mock-infected conditions for n ≥ 2 donors are shown. (D) Histograms depict CD69 expression on NK cells for one exemplary donor after 48 h coculture with infected HT-29 or 48 h post inoculation for NK cell only conditions. (E) CD69 MFI data for n = 4 donors from two experiments are summarized.

Supplementary Figure 2

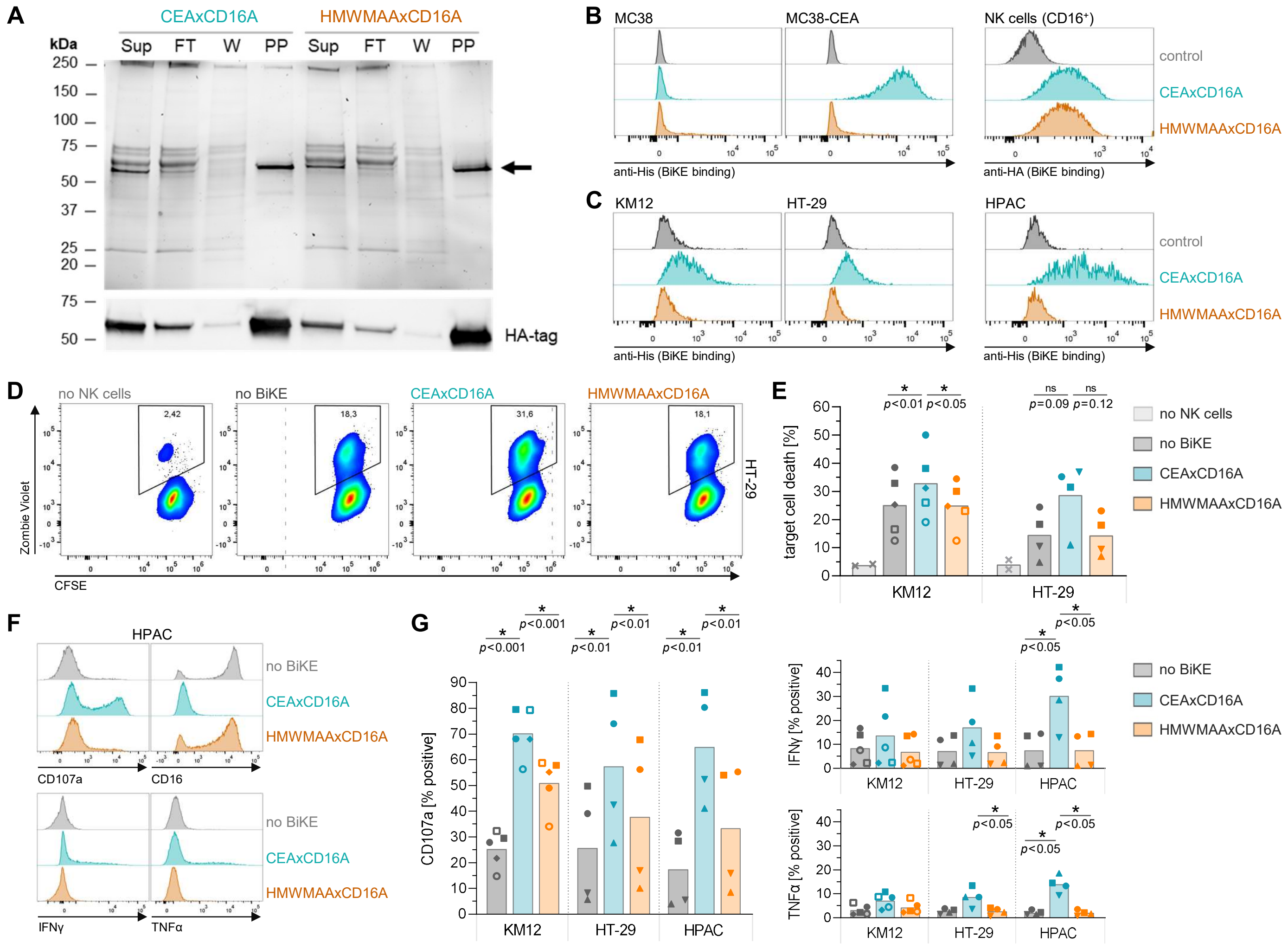

Functionality of MV-encoded bispecific killer engagers

(A) Purification of BiKE proteins. Supernatants of MV-BiKE infected Vero cells cultured in serum-free medium were collected for purification of the respective CEA or HMWMAA-targeting BiKE (vBiKE). Proteins were purified by affinity exchange chromatography via the His_6 tag. Fractions were analyzed by polyacrylamide gel electrophoresis. Equal volumes were loaded except for the purified protein samples, which were adjusted to 2 µg total protein. Stain-free imaging of the gel (top) was performed prior to western blotting (bottom). BiKE proteins were detected via anti-HA biotin and streptavidin-HRP. Sup, supernatant of MV-BiKE infected cells; FT, column flow-through; W, wash fraction; PP, purified protein. Arrow indicates BiKE protein bands (57.5 kDa). (B) BiKE binding to CEA-expressing MC38 cells, $CD16^+$ NK cells and (C) colorectal cancer (KM12, HT-29) or pancreatic ductal adenocarcinoma (HPAC) cell lines. Cells were incubated with respective vBiKEs and analyzed by flow cytometry. Antibodies specific for the His_6 or HA tag were used to detect BiKE binding to CEA on tumor cells or to CD16 on NK cells, respectively.

Legend for panels (D) – (G) continued on next page.

Supplementary Figure 2 (continued)

(D) and (E) Efficacy of vBiKEs against colorectal cancer cells. CFSE-labeled tumor cells were cocultured with NK cells (E:T = 5:1) and vBiKEs (10 ng/µL). Target cell-specific CEAxCD16A BiKEs are compared to HMWMAAxCD16A BiKEs with irrelevant specificity. Target cell killing was analyzed by flow cytometry based on Zombie Violet staining. (D) Exemplary pseudocolor plots for HT-29 illustrate separation of live and dead $CFSE^+$ tumor cell populations. (E) Mean target cell death is shown for $n \geq 4$ donors per cell line with different symbols to discriminate individual donors. (F) and (G) NK cell effector functions in response to vBiKE. (F) Exemplary histograms for HPAC target cells illustrate NK cell degranulation (CD107a), CD16 surface levels and intracellular cytokine accumulation (IFNγ and TNFα). (G) Mean percentages of $CD107a^+$, IFNγ^+ and TNFα^+ NK cells are summarized for $n \geq 4$ donors per cell line with symbols as in (E). Statistical analysis was performed by one way ANOVA with Šidák's multiple comparisons test.

Supplementary Figure 3

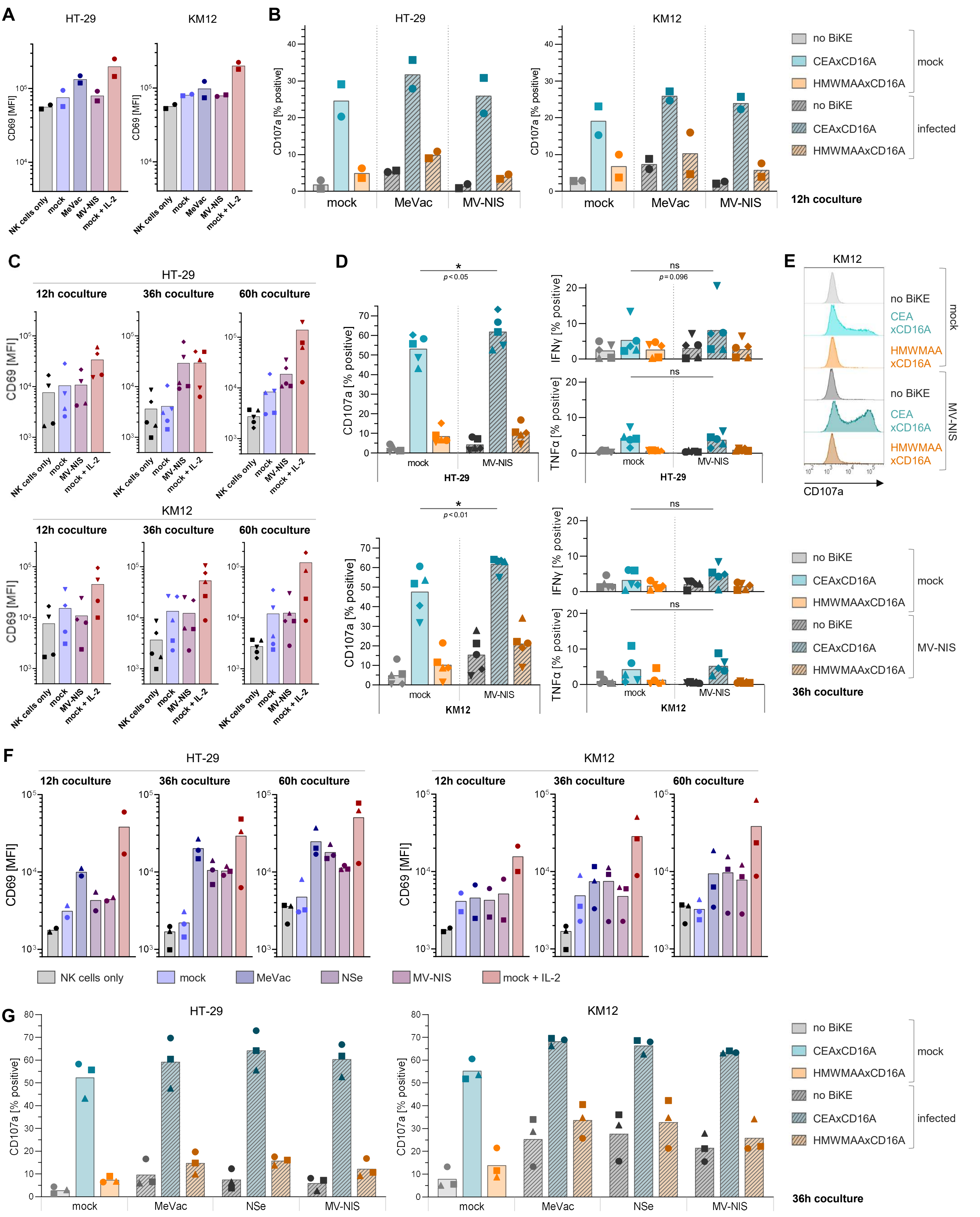

NK cell activation and degranulation in MV plus BiKE combination therapy with lab-grade and highly purified virus preparations

See next page for Figure Legend.

Supplementary Figure 3

HT-29 and KM12 colorectal cancer cells were inoculated with lab-grade MeVac or NSe and highly purified MV-NIS at MOI 1. NK cells were added 24 h post inoculation. Firstly, CD69 expression on NK cells after exposure to the infected cells was analyzed at different time points post inoculation. Secondly, non-infected target cells and vBiKEs were added to the coculture at designated time points and incubated for 4 h until NK cell degranulation was analyzed (compare Figure 1A). (A) CD69 MFI on NK cells was analyzed after 12 h co-incubation with infected HT-29 or KM12, i.e. 36 h post inoculation. (B) Quantification of NK cell degranulation is shown for vBiKE treatment combined with the exposure to infected HT-29 and KM12, with cocultures started 12 h prior to vBiKE addition. (C) NK cells were co-incubated with HT-29 or KM12 cells infected with MV-NIS or subjected to mock infection. CD69 expression was analyzed at multiple time points as indicated. (D) – (E) After 36 h coculture, i.e. 60 h post inoculation, vBiKE treatment was performed and the percentages of CD107a$^+$, IFNγ^+, and TNFα^+ NK cells were determined 4 h after treatment. (D) Percentages of NK cells positive for the degranulation marker CD107a and cytokine expression for n = 5 donors and (E) exemplary histograms are shown. (F) CD69 expression on NK cells in coculture with infected HT-29 and KM12 cells was analyzed for lab-grade MeVac and NSe and highly purified MV-NIS at designated time points post inoculation. MFI is shown for n = 2 donors (12 h) or n = 3 donors (36 h, 60 h). (G) After 36 h coculture with MeVac, NSe, or MV-NIS infected tumor cells, i.e. 60 h post inoculation, vBiKE treatment was performed, and NK cells were analyzed 4 h after treatment. Percentages of NK cells positive for the degranulation marker CD107a are depicted.

Data for mock and MeVac conditions displayed in (A) are also included in the summary bar plots in Figure 1C. Experiments in (F) and (G) were performed in parallel with (C) – (E) for a subset of donors, so that data for mock and MV-NIS as well as NK cell only and IL-2 controls is displayed twice using identical symbols per donor. Statistical analysis in D was performed by paired t-test.

Supplementary Figure 4

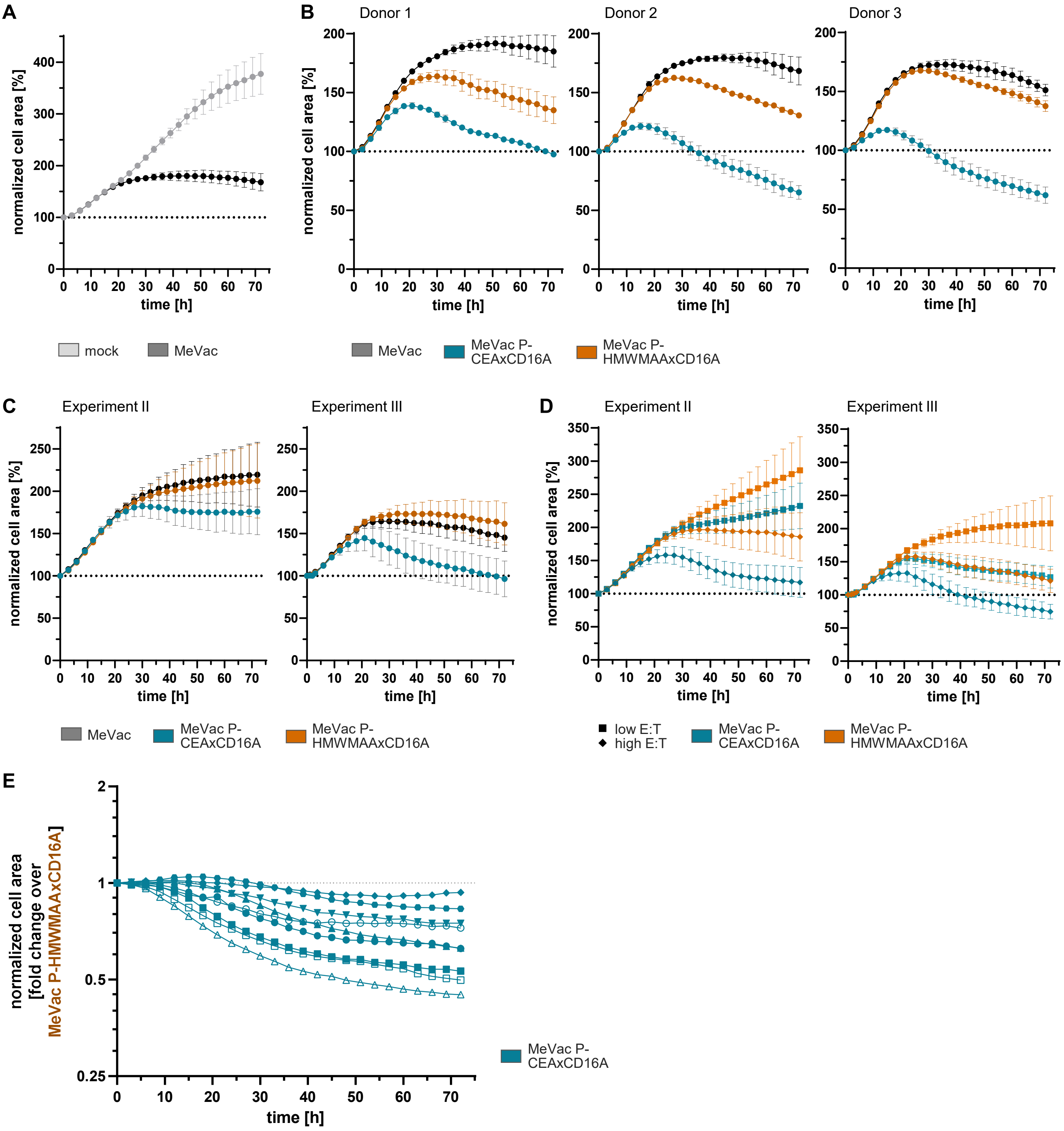

Live-cell imaging analysis of MV-BiKE immunovirotherapy against bystander cancer cells

In vitro coculture experiments were performed as illustrated in Figure 3A. Non-infected HT-29 colorectal cancer cells transduced with tagRFP were cocultured with MV-BiKE infected HT-29 cells and NK cells. The tagRFP^{+} tumor cell area was monitored for 72h using live-cell imaging. Exemplary time course plots show the kinetics of bystander tumor cell area during coculture.

Continued on next page.

Supplementary Figure 4 (continued)

(A) Comparison of mock-infected conditions and MV without transgene. Mean and SD for three donors in the same experiment as depicted in Figure 3F are shown. (B) For each donor summarized in Figure 3F, individual plots with mean and SD of three technical replicates are depicted. (C) and (D) Time course plots illustrate effects of MV-BiKE treatment at different E:T ratios as in Figure 3F and 3H, respectively. In each plot, mean and SD of three donors in one independent experiment are depicted. Quantification and statistical analysis of all three independent experiments is summarized in Figure 3G and 3I. (E) Time course plot depicting normalized cell area of HT-29 bystander cells in MeVac P-CEAxCD16A-treated cocultures. Fold change over conditions with MV encoding irrelevant HMWMAAxCD16A BiKE is summarized for n = 9 donors.

Supplementary Figure 5

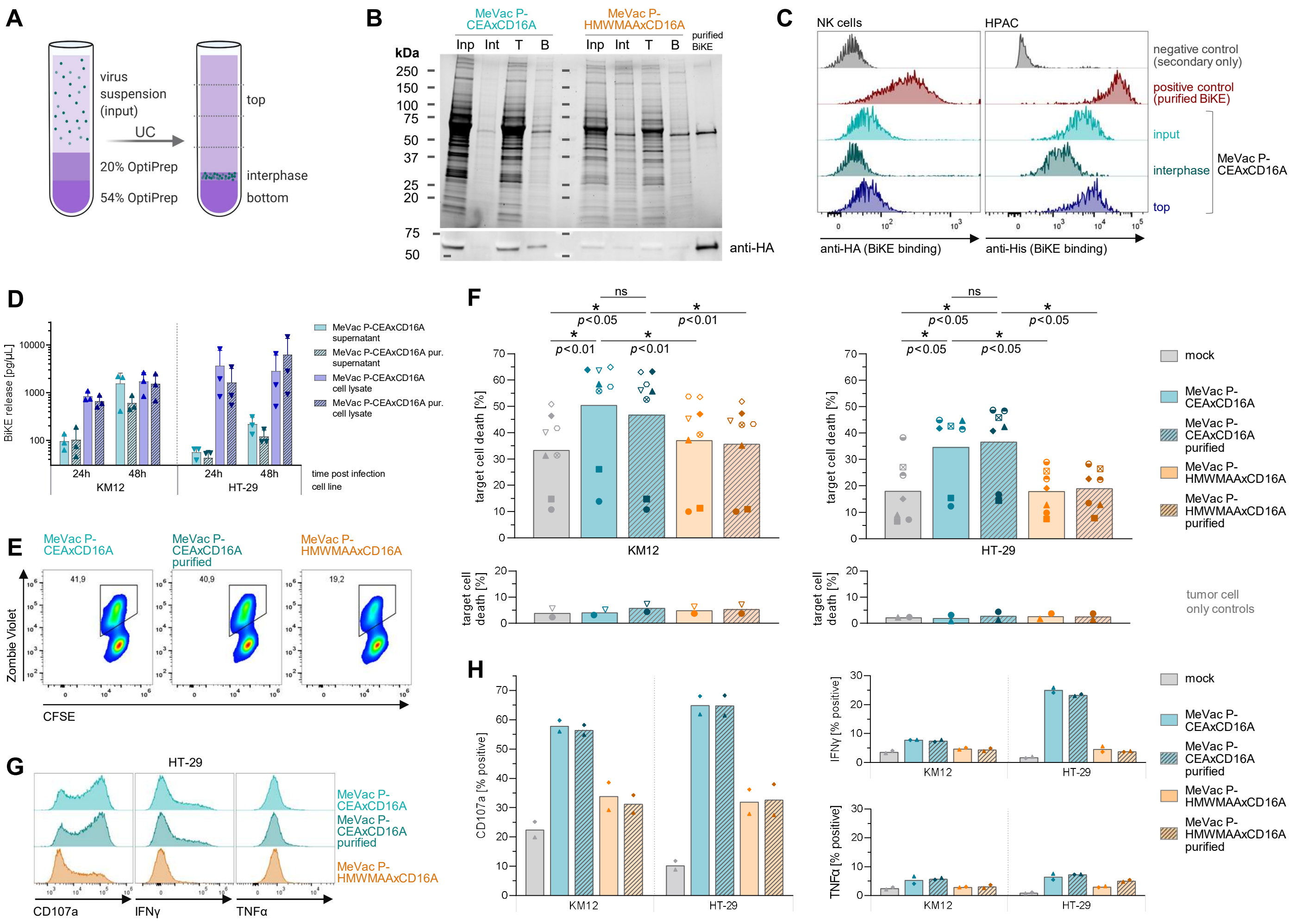

Efficacy of immunovirotherapy with MV-BiKE purified via ultracentrifugation

(A) Schematic representation of MV purification via OptiPrep fractionation ultracentrifugation (UC). The virus suspension was layered onto 54% and 20% OptiPrep phases and, following ultracentrifugation, the interphase containing purified viruses was collected. In addition, the bottom and top fractions were kept for analysis. (B) Total protein and BiKE levels in respective samples were analyzed by polyacrylamide gel electrophoresis, stain-free imaging of the gel (top) and western blot (bottom). For the input virus suspension (Inp), purified virus interphase (Int) and bottom fraction (B), 5×10^4 ciu were loaded per lane. For the top fraction (T), the same sample volume as for the input was loaded for direct comparison. Purified CEAxCD16A vBiKE (1 µg) served as a control. The HA-tagged BiKE proteins were detected with anti-HA biotin and streptavidin-HRP.

Legend for panels (C) – (H) continued on next page.

Supplementary Figure 5 (continued)

(C) CEAxCD16A BiKE binding to $CD16^+$ NK cells and HPAC tumor cells after incubation with designated fractions from the purification was assessed by flow cytometry. For the input and interphase fractions, volumes corresponding to 5×10^4 ciu were used for incubation. For the top fraction, the same volume as for the input was applied. BiKE binding to CD16 on NK cells and to CEA on cancer cells was detected with anti-HA and anti-His_6 antibodies, respectively. (D) BiKE expression after infection with purified virus. KM12 and HT-29 cells were inoculated with conventional lab-grade MeVac P-CEAxCD16A compared to UC-purified virus at MOI 1. Supernatants and cell lysates were collected at indicated time points. BiKE concentrations were determined via ELISA with purified vBiKEs as standard. Error bars indicate SD. (E) - (H) Efficacy of purified MV-BiKE in comparison to lab-grade counterparts. MV-BiKE infected colorectal cancer cells were cocultured with CFSE-labeled, non-infected tumor cells and IL-2 pre-stimulated NK cells for 12 h. NK cell degranulation, cytokine expression and killing of $CSFE^+$ target cells were analyzed by flow cytometry (compare Figure 3A). (E) Exemplary pseudocolor plots for HT-29 depict $CFSE^+$ target cell viability. (F) Mean bystander target cell death per treatment is shown for n = 8 donors (KM12, left) and n = 7 donors (HT-29, right) with individual donors represented by different symbols. Tumor cell only controls without NK effector cells are shown below the respective bar plots. (G) Exemplary histograms depict NK cell degranulation (CD107a) and intracellular cytokine accumulation for HT-29 target cells. (H) Mean percentages of $CD107a^+$, $IFN\gamma^+$ and $TNF\alpha^+$ NK cells are summarized for n = 2 donors per cell line with symbols for individual donors as in (F).

Data for mock and the non-purified MeVac P-BiKE conditions are also included in the summary bar plots in Figure 3C and displayed again for comparison. Statistical analysis was performed by one way ANOVA with Šidák's multiple comparisons test.

Supplementary Figure 6

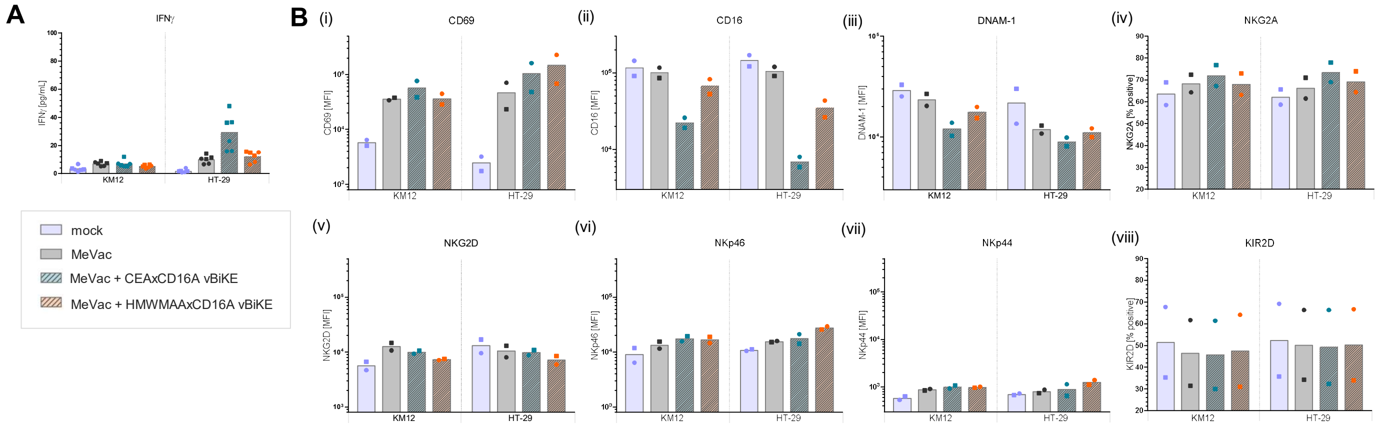

NK cell characterization in response to MV-BiKE therapy

NK cell surface markers and IFNγ release were analyzed after coculture with infected colorectal cancer cells as in Figure 5. MeVac infection was compared to NK cell only controls and combination treatment with MV and purified vBiKEs (10 ng/μL). (A) IFNγ concentrations in coculture supernatants were quantified by ELISA. Results are shown for KM12 and HT-29 tumor cells with technical triplicates for n = 2 donors, represented by different symbols. (B) NK cell surface markers were characterized by flow cytometry. Mean fluorescence intensity (MFI) or the percentage of cells expressing the respective marker are summarized for n = 2 donors. Different symbols represent individual donors. For CD16, the mean of three technical replicates is shown per donor. Experiment was performed in parallel with experiments shown in Figure 4B and 4C for a subset of donors as indicated by respective symbols. Mock conditions are displayed again for direct comparison.

Supplementary Figure 7

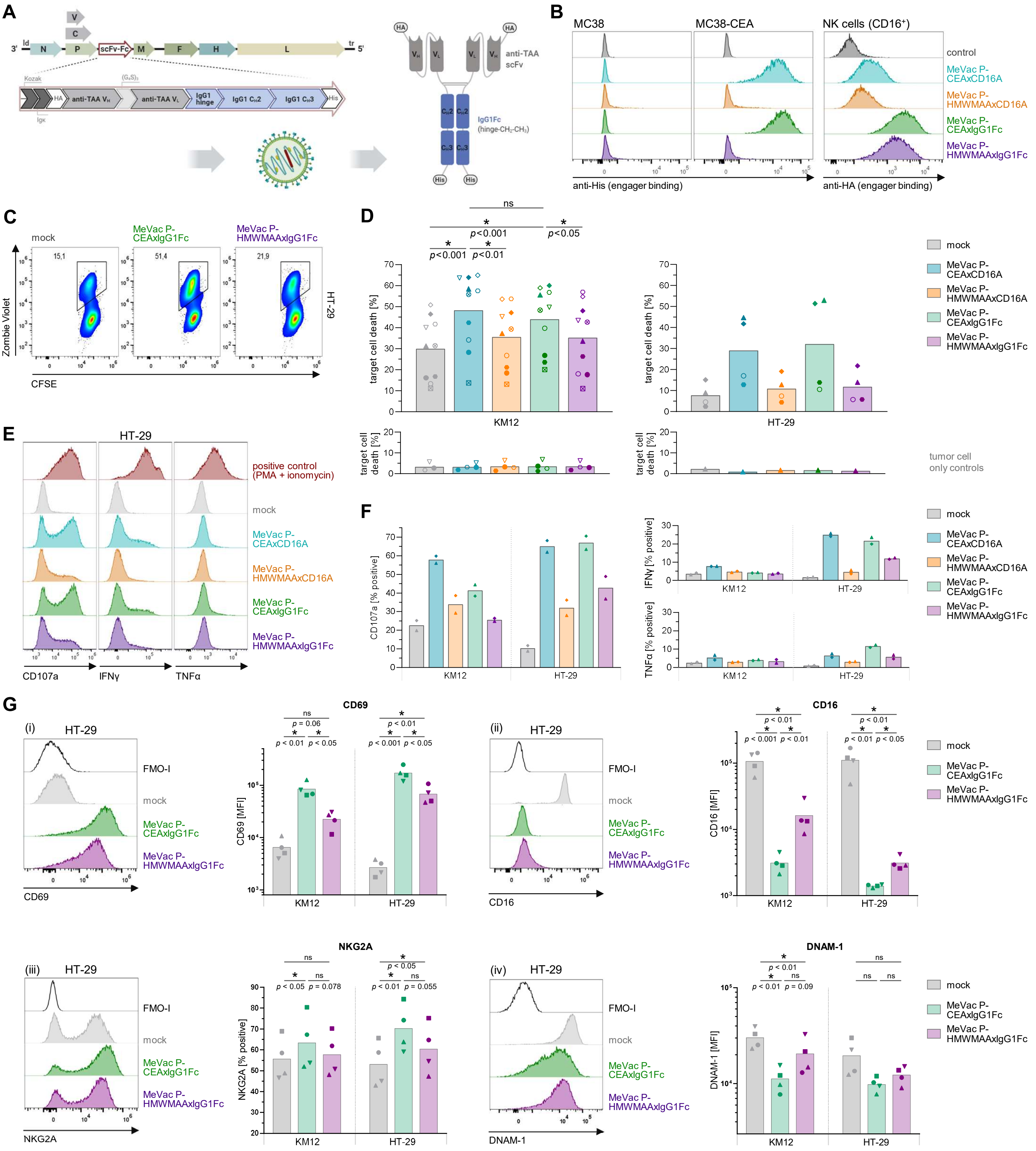

Comparison of MV-encoded BiKEs and scFv-Fcs for NK cell engagement

See next page for Figure Legend.

Supplementary Figure 7

(A) Schematic of the recombinant MV encoding a scFv-Fc protein. Analogous to MV-BiKE, the transgene is encoded downstream of the *P* open reading frame, with hemagglutinin (HA) and hexa histidine (His_6) tags included for detection as well as Kozak and Igκ leader sequences added for translation and secretion. The transgene-encoding virus can be rescued via a reverse genetics system and results in release of dimerized scFv-Fc proteins from infected cells. C_H, constant heavy chain; $(G_4S)_3$, glycine-serine peptide linker; scFv, single chain variable fragment with variable heavy (V_H) and variable light (V_L) chain; TAA, tumor-associated antigen. (B) Binding of MV-encoded scFv-Fc to tumor and effector cells. Cells were incubated with respective virus suspensions and analyzed by flow cytometry. Antibodies specific for the His_6 or HA tag were used to detect BiKE binding to CEA on tumor cells or to Fc receptors on NK cells, respectively. For MC38 and MC38-CEA, controls were stained with anti-His_6 antibody only. For NK cells, MeVac without transgene with subsequent anti-HA antibody staining was used as control. (C) - (F) Efficacy of MV-scFv-Fc in comparison to MV-BiKE and mock-infected control. Infected colorectal cancer cells were co-incubated with non-infected, CFSE-labeled tumor cells and IL-2 pre-stimulated NK cells for 12 h. Killing of $CSFE^+$ target cells, NK cell degranulation and cytokine expression were analyzed by flow cytometry (compare Figure 3A). (C) Pseudocolor plots for one exemplary donor with HT-29 target cells illustrate $CFSE^+$ tumor cell viability. (D) Target cell death is summarized for n = 10 donors (KM12, left) and n = 4 donors (HT-29, right) for each treatment with symbols representing individual donors. Controls without NK effector cells are shown below. (E) Histograms depict NK cell degranulation (CD107a) and intracellular cytokine accumulation (IFNγ, TNFα) for HT-29 target cells treated with CEA-targeting MV-BiKE or MV-scFv-Fc and respective controls. (F) Mean percentages of $CD107a^+$, IFNγ^+ and TNFα^+ NK cells are shown for n = 2 donors per cell line with donors represented by symbols as in (D). Data for mock and the MeVac P-BiKE conditions are also included in the summary bar plots in Figure 3C, 3E and Figure S5 and displayed again for comparison. Statistical analysis was performed by one way ANOVA with Šidák's multiple comparisons test. (G) Characterization of NK cell surface markers in response to MV-scFv-Fc treatment. Tumor cells were inoculated at MOI 1 one day prior to coculture. Freshly isolated NK cells were co-incubated with infected cells for 48 h and surface markers on NK cells were analyzed by flow cytometry (compare Figure 4A). For each marker, exemplary histograms (left) and a summary of either the MFIs or the percentage of cells expressing the respective marker (right) are shown for n = 4 donors. Different symbols represent individual donors. For CD16, mean of three technical replicates is shown per donor. FMO-I, Fluorescence Minus One + Isotype control. Statistical analysis was performed by one way ANOVA with Šidák's multiple comparisons test.

Supplementary Figure 8

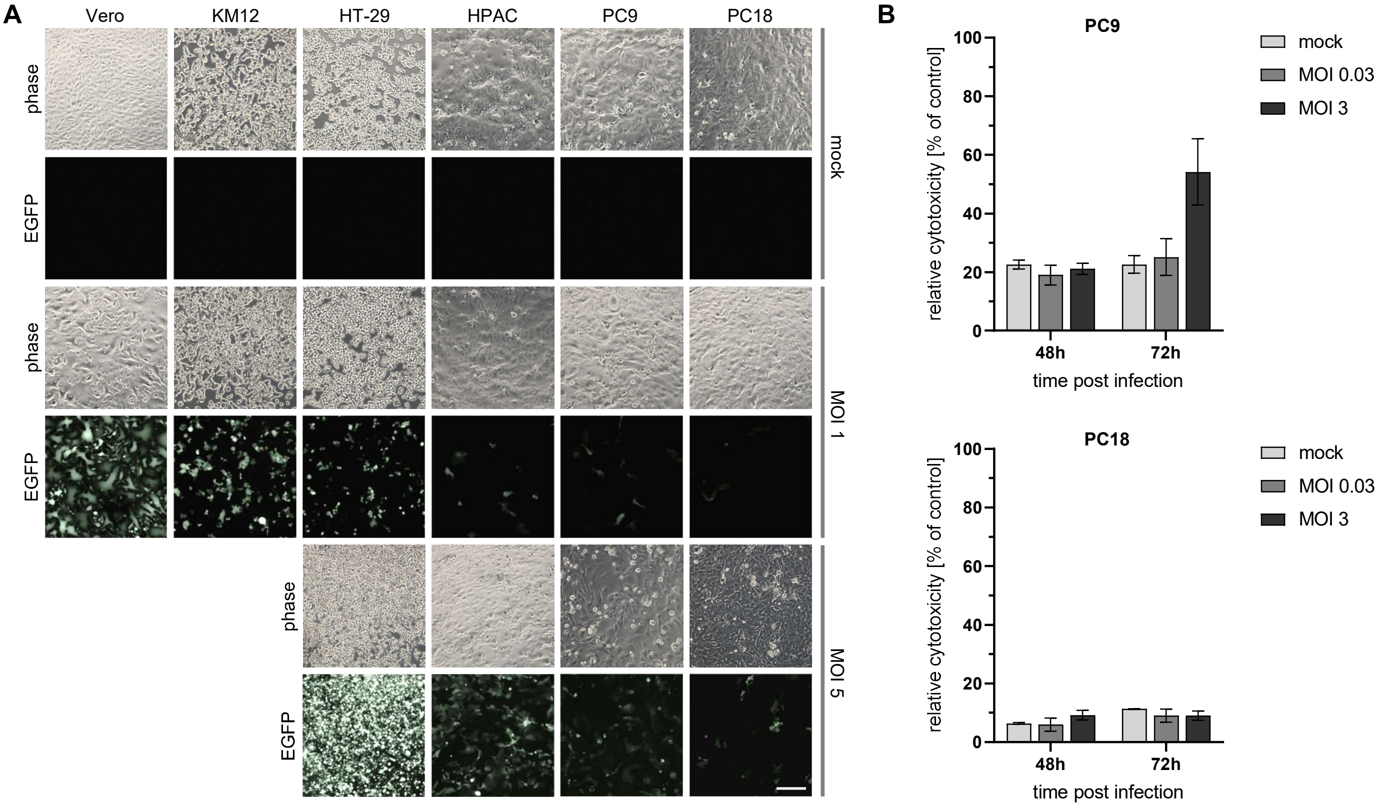

Cancer cell sensitivity towards MV infection

Sensitivity of patient-derived pancreatic cancer short term cultures (PC9, PC18) towards MV infection in comparison to Vero producer cells and colorectal cancer (KM12, HT-29) or pancreatic adenocarcinoma (HPAC) cell lines. (A) Cells were inoculated with MeVac ldEGFP at indicated MOIs. Images were acquired 36 h post infection. Scale bar, 200 µm (B) Patient-derived cultures were inoculated with highly purified MV-NIS at MOI 0.03 and MOI 3, or mock-infected with medium only. Relative cytotoxicity was calculated based on LDH release.

Supplementary Figure 9

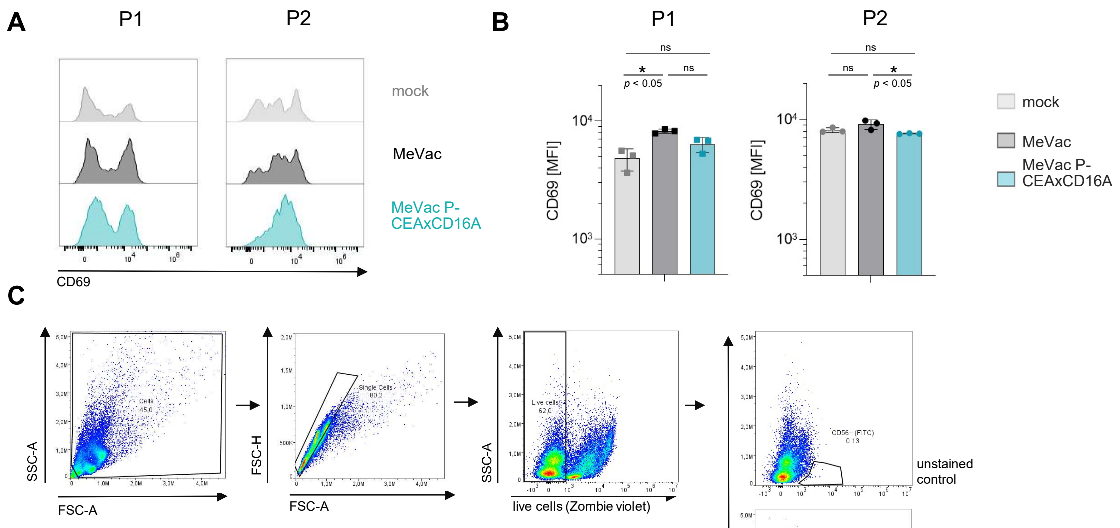

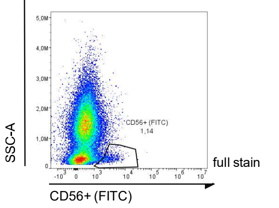

D

Samples	CD56+ cell counts	
	Patient 1	Patient 2
mock_1	291	200
mock_2	84	231
mock_3	307	125
MeVac_1	1576	434
MeVac_2	1231	568
MeVac_3	1016	411
MeVac-P-CEAxCD16A_1	2208	1582
MeVac-P-CEAxCD16A_2	2051	1498
MeVac-P-CEAxCD16A_3	2068	1785

MV-BiKE activity in primary colorectal cancer.

NK cell activation was analyzed after infection of single cell suspensions of patient-derived colorectal carcinoma specimens. MV-BiKE infections were compared to MV infection and mock controls. (A) NK cell activation (CD69) in response to MV-BiKE therapy is depicted in exemplary histograms. (B) CD69 mean fluorescence intensity (MFI) is summarized for n = 3 technical replicates of n = 2 patients (P1, left; P2, right) with different symbols representing individual patients. (C) Gating strategy of flow cytometry analysis. (D) Cell counts of $CD56^{+}$ cells in flow cytometry data set are listed for patients P1 and P2. Statistical analysis was performed by one way ANOVA with Šidák's multiple comparisons test.
