# Supplementary material for: A vector-encoded bispecific killer engager to harness virus-activated NK cells as anti-tumor effectors
Source: Cell Death Dis. 2023 Feb 10;14(2):104. doi: 10.1038/s41419-023-05624-3 (PMC9918448; doi:10.1038/s41419-023-05624-3)
Supplement: Supplementary file 3 — Authorship confirmations [file 41419_2023_5624_MOESM3_ESM.pdf]

Re: Manuscript MV BiKE CDDIS-22-3819: Accepted in Principle

Flörchinger, Alessia

Tue 1/24/2023 11:47 PM

To:Ungerechts, Guy <guy.ungerechts@nct-heidelberg.de>; Engeland, Christine <christine.engeland@nct-heidelberg.de>;

Cc:Schäfer, Theresa <theresa.schaefer@dkfz-heidelberg.de>; gwen.fuchs@gmx.de <gwen.fuchs@gmx.de>; k.chester@ucl.ac.uk <k.chester@ucl.ac.uk>; Jäger, Dirk <dirk.jaeger@nct-heidelberg.de>; Ball, Claudia <claudia.ball@nct-dresden.de>; Jessica Klein <jessica.e.klein@web.de>; Finkbeiner, Maximiliane <Maximiliane.Finkbeiner@uni-wh.de>;

I confirm.

Best regards,
Alessia

From: Ungerechts, Guy <guy.ungerechts@nct-heidelberg.de>
Sent: Tuesday, January 24, 2023 8:49:46 PM
To: Engeland, Christine <christine.engeland@nct-heidelberg.de>
Cc: Schäfer, Theresa <theresa.schaefer@dkfz-heidelberg.de>; gwen.fuchs@gmx.de <gwen.fuchs@gmx.de>; k.chester@ucl.ac.uk <k.chester@ucl.ac.uk>; Jäger, Dirk <dirk.jaeger@nct-heidelberg.de>; Ball, Claudia <claudia.ball@nct-dresden.de>; Flörchinger, Alessia <a.floerchinger@dkfz-heidelberg.de>; Jessica Klein <jessica.e.klein@web.de>; Finkbeiner, Maximiliane <Maximiliane.Finkbeiner@uni-wh.de>
Subject: Re: Manuscript MV BiKE CDDIS-22-3819: Accepted in Principle

Confirmed.

Best,
Guy

Prof. Dr. med. Dr. rer. nat. Guy Ungerechts
Stellv. Ärztlicher Direktor
Leitender Oberarzt
Abteilung Medizinische Onkologie
Nationales Centrum für Tumorerkrankungen (NCT) Heidelberg
Universitätsklinikum Heidelberg
Im Neuenheimer Feld 460
D-69120 Heidelberg
Germany

Tel.: +49 6221 567229
Fax: +49 6221 565218
Email: guy.ungerechts@nct-heidelberg.de

> Am 24.01.2023 um 18:52 schrieb Engeland, Christine <christine.engeland@nct-heidelberg.de>:

> Dear Colleagues,
>
> Our collaborators from Helios University Clinic Wuppertal Drs.

Johannes Dörner, Hubert Zirngibl, Maximilian Ackermann, Hans M. Kvasnicka

contributed to the experiments performed during the revision of our manuscript.

Please confirm that you agree that they are included as co-authors by responding to this email.

Thank you.

Best regards,

Christine

From: cddisease@springernature.com <cddisease@springernature.com>
Sent: Tuesday, January 24, 2023 3:46 PM
To: Engeland, Christine
Subject: CDDIS-22-3819RR Initial Quality Check

Dear Professor Engeland,

In checking in your manuscript submitted to Cell Death & Disease it has come to our attention that the following must be addressed before we can proceed.

1. It has come to our attention that your most recent author list differs from the one in your original submission. We find that the following authors have been added since your initial submission: Johannes Dörner; Hubert Zirngibl; Maximilian Ackermann; Hans M. Kvasnicka.

Please request agreement from all authors including additions and deletions, these can be collected in the following way:

Email your co-authors with the change, and ask them to reply to your email confirming that they agree to these changes. Once you have collected these replies, please combine all of the co-authors' email responses in one document and upload this file to your submission.

Your paper is now available for you to edit, you may access via the following link:

https://mts-cddis.nature.com/cgi-bin/main.plex?el=A2CA3ZRG4C6CMuZ6F1A9ftdTUgP0sjec20MwrNt08A2gZ

(Press/Click on the above link to be automatically sent to the web page.)

Please make the correction(s) as specified above and resubmit your paper following the same steps as before.

If you have any questions please feel free to contact us.

Sincerely,

Editorial Office
Cell Death & Disease
cddisease@springernature.com

This email has been sent through the NPG Manuscript Tracking System NY-610A-NPG&MTS

Confidentiality Statement:

This e-mail is confidential and subject to copyright. Any unauthorised use or disclosure of its contents is prohibited. If you have received this email in error please notify our Manuscript Tracking System Helpdesk team at http://platformsupport.nature.com .

Details of the confidentiality and pre-publicity policy may be found here http://www.nature.com/authors/policies/confidentiality.html

Privacy Policy | Update Profile

Re: Manuscript MV BiKE CDDIS-22-3819: Accepted in Principle

Jessica Klein <jessica.e.klein@web.de>

Wed 1/25/2023 7:15 AM

To: Flörchinger, Alessia <a.floerchinger@dkfz-heidelberg.de>; Ungerechts, Guy <guy.ungerechts@nct-heidelberg.de>; Engeland, Christine <christine.engeland@nct-heidelberg.de>;

Cc: Schäfer, Theresa <theresa.schaefer@dkfz-heidelberg.de>; gwen.fuchs@gmx.de <gwen.fuchs@gmx.de>; k.chester@ucl.ac.uk <k.chester@ucl.ac.uk>; Jäger, Dirk <dirk.jaeger@nct-heidelberg.de>; Ball, Claudia <claudia.ball@nct-dresden.de>; Finkbeiner, Maximiliane <Maximiliane.Finkbeiner@uni-wh.de>;

I confirm.

All the best,

Jessica

From: Flörchinger, Alessia <a.floerchinger@dkfz-heidelberg.de>
Sent: Tuesday, January 24, 2023 11:48 PM
To: Ungerechts, Guy <guy.ungerechts@nct-heidelberg.de>; Engeland, Christine <christine.engeland@nct-heidelberg.de>
Cc: Schäfer, Theresa <theresa.schaefer@dkfz-heidelberg.de>; gwen.fuchs@gmx.de <gwen.fuchs@gmx.de>; k.chester@ucl.ac.uk <k.chester@ucl.ac.uk>; Jäger, Dirk <dirk.jaeger@nct-heidelberg.de>; Ball, Claudia <claudia.ball@nct-dresden.de>; Jessica Klein <jessica.e.klein@web.de>; Finkbeiner, Maximiliane <Maximiliane.Finkbeiner@uni-wh.de>
Subject: Re: Manuscript MV BiKE CDDIS-22-3819: Accepted in Principle

I confirm.

Best regards,
Alessia

From: Ungerechts, Guy <guy.ungerechts@nct-heidelberg.de>
Sent: Tuesday, January 24, 2023 8:49:46 PM
To: Engeland, Christine <christine.engeland@nct-heidelberg.de>
Cc: Schäfer, Theresa <theresa.schaefer@dkfz-heidelberg.de>; gwen.fuchs@gmx.de <gwen.fuchs@gmx.de>; k.chester@ucl.ac.uk <k.chester@ucl.ac.uk>; Jäger, Dirk <dirk.jaeger@nct-heidelberg.de>; Ball, Claudia <claudia.ball@nct-dresden.de>; Flörchinger, Alessia <a.floerchinger@dkfz-heidelberg.de>; Jessica Klein <jessica.e.klein@web.de>; Finkbeiner, Maximiliane <Maximiliane.Finkbeiner@uni-wh.de>
Subject: Re: Manuscript MV BiKE CDDIS-22-3819: Accepted in Principle

Confirmed.

Best,
Guy

Prof. Dr. med. Dr. rer. nat. Guy Ungerechts
Stellv. Ärztlicher Direktor
Leitender Oberarzt
Abteilung Medizinische Onkologie
Nationales Centrum für Tumorerkrankungen (NCT) Heidelberg
Universitätsklinikum Heidelberg
Im Neuenheimer Feld 460

D-69120 Heidelberg
Germany

Tel.: +49 6221 567229
Fax: +49 6221 565218
Email: guy.ungerechts@nct-heidelberg.de

> Am 24.01.2023 um 18:52 schrieb Engeland, Christine <christine.engeland@nct-heidelberg.de>:

> Dear Colleagues,
>
> Our collaborators from Helios University Clinic Wuppertal Drs.
>
> Johannes Dörner, Hubert Zirngibl, Maximilian Ackermann, Hans M. Kvasnicka
>
> contributed to the experiments performed during the revision of our manuscript.
>
> Please confirm that you agree that they are included as co-authors by responding to this email.
>
> Thank you.
>
> Best regards,
>
> Christine
>
> ---
>
> **From:** cddisease@springernature.com <cddisease@springernature.com>
> **Sent:** Tuesday, January 24, 2023 3:46 PM
> **To:** Engeland, Christine
> **Subject:** CDDIS-22-3819RR Initial Quality Check
>
> Dear Professor Engeland,
>
> In checking in your manuscript submitted to Cell Death & Disease it has come to our attention that the following must be addressed before we can proceed.
>
> 1. It has come to our attention that your most recent author list differs from the one in your original submission. We find that the following authors have been added since your initial submission: Johannes Dörner; Hubert Zirngibl; Maximilian Ackermann; Hans M. Kvasnicka.
>
> Please request agreement from all authors including additions and deletions, these can be collected in the following way:
>
> Email your co-authors with the change, and ask them to reply to your email confirming that they agree to these changes. Once you have collected these replies, please combine all of the co-authors' email responses in one document and upload this file to your submission.

Your paper is now available for you to edit, you may access via the following link:

https://mts-cddis.nature.com/cgi-bin/main.plex?el=A2CA3ZRG4C6CMuZ6F1A9ftdTUgP0sjec20MwrNt08A2gZ

(Press/Click on the above link to be automatically sent to the web page.)

Please make the correction(s) as specified above and resubmit your paper following the same steps as before.

If you have any questions please feel free to contact us.

Sincerely,

Editorial Office
Cell Death & Disease
cddisease@springernature.com

This email has been sent through the NPG Manuscript Tracking System NY-610A-NPG&MTS

Confidentiality Statement:

This e-mail is confidential and subject to copyright. Any unauthorised use or disclosure of its contents is prohibited. If you have received this email in error please notify our Manuscript Tracking System Helpdesk team at http://platformsupport.nature.com .

Details of the confidentiality and pre-publicity policy may be found here http://www.nature.com/authors/policies/confidentiality.html

Privacy Policy | Update Profile

AW: Manuscript MV BiKE CDDIS-22-3819: Accepted in Principle

Finkbeiner, Maximiliane <Maximiliane.Finkbeiner@uni-wh.de>

Tue 1/24/2023 7:19 PM

To:Engeland, Christine <christine.engeland@nct-heidelberg.de>;

Dear Christine,

I hereby confirm that I agree to the changes.

Best regards,
Maxi

Von: Engeland, Christine <christine.engeland@nct-heidelberg.de>
Gesendet: Dienstag, 24. Januar 2023 18:52
An: Schäfer, Theresa; gwen.fuchs@gmx.de; k.chester@ucl.ac.uk; Jäger, Dirk; Ball, Claudia; Ungerechts, Guy
Cc: Flörchinger, Alessia; Jessica Klein; Finkbeiner, Maximiliane
Betreff: Re: Manuscript MV BiKE CDDIS-22-3819: Accepted in Principle

Dear Colleagues,

Our collaborators from Helios University Clinic Wuppertal Drs.
Johannes Dörner, Hubert Zirngibl, Maximilian Ackermann, Hans M. Kvasnicka
contributed to the experiments performed during the revision of our manuscript.

Please confirm that you agree that they are included as co-authors by responding to this email.

Thank you.
Best regards,
Christine

From: cddisease@springernature.com <cddisease@springernature.com>
Sent: Tuesday, January 24, 2023 3:46 PM
To: Engeland, Christine
Subject: CDDIS-22-3819RR Initial Quality Check

Dear Professor Engeland,

In checking in your manuscript submitted to Cell Death & Disease it has come to our attention that the following must be addressed before we can proceed.

1. It has come to our attention that your most recent author list differs from the one in your original submission. We find that the following authors have been added since your initial submission: Johannes Dörner; Hubert Zirngibl; Maximilian Ackermann; Hans M. Kvasnicka.

Please request agreement from all authors including additions and deletions, these can be collected in the following way:

Email your co-authors with the change, and ask them to reply to your email confirming that they

agree to these changes. Once you have collected these replies, please combine all of the co-authors' email responses in one document and upload this file to your submission.

Your paper is now available for you to edit, you may access via the following link:

https://mts-cddis.nature.com/cgi-bin/main.plex?el=A2CA3ZRG4C6CMuZ6F1A9ftdTUgP0sjec20MwrNt08A2gZ

(Press/Click on the above link to be automatically sent to the web page.)

Please make the correction(s) as specified above and resubmit your paper following the same steps as before.

If you have any questions please feel free to contact us.

Sincerely,

Editorial Office
Cell Death & Disease
cddisease@springernature.com

This email has been sent through the NPG Manuscript Tracking System NY-610A-NPG&MTS

Confidentiality Statement:

This e-mail is confidential and subject to copyright. Any unauthorised use or disclosure of its contents is prohibited. If you have received this email in error please notify our Manuscript Tracking System Helpdesk team at http://platformsupport.nature.com .
Details of the confidentiality and pre-publicity policy may be found here http://www.nature.com/authors/policies/confidentiality.html
Privacy Policy | Update Profile

Private Universität Witten/Herdecke gGmbH
Alfred-Herrhausen-Straße 50
D - 58448 Witten

Homepage: www.uni-wh.de

Geschäftsführung: Prof. Dr. Martin Butzlaff (Präsident), Dipl. oec. Jan Peter Nonnenkamp (Kanzler)

Sitz der Gesellschaft: Witten
Handelsregister des Amtsgerichts Bochum Nr. HRB 8671

Re: Manuscript MV BiKE CDDIS-22-3819: Accepted in Principle

Schäfer, Theresa

Wed 1/25/2023 9:41 AM

To:Engeland, Christine <christine.engeland@nct-heidelberg.de>;

Cc:gwen.fuchs@gmx.de <gwen.fuchs@gmx.de>; k.chester@ucl.ac.uk <k.chester@ucl.ac.uk>; Jäger, Dirk <dirk.jaeger@nct-heidelberg.de>; Ball, Claudia <claudia.ball@nct-dresden.de>; Ungerechts, Guy <guy.ungerechts@nct-heidelberg.de>; Flörchinger, Alessia <a.floerchinger@dkfz-heidelberg.de>; Jessica Klein <jessica.e.klein@web.de>; Finkbeiner, Maximiliane <Maximiliane.Finkbeiner@uni-wh.de>;

I confirm.

Best,
Theresa

> Le 24.01.2023 à 18:52, Engeland, Christine <christine.engeland@nct-heidelberg.de> a écrit :

> Dear Colleagues,
>
> Our collaborators from Helios University Clinic Wuppertal Drs.
>
> Johannes Dörner, Hubert Zirngibl, Maximilian Ackermann, Hans M. Kvasnicka
>
> contributed to the experiments performed during the revision of our manuscript.
>
> Please confirm that you agree that they are included as co-authors by responding to this email.
>
> Thank you.
>
> Best regards,
>
> Christine
>
> ---
>
> **From:** cddisease@springernature.com <cddisease@springernature.com>
> **Sent:** Tuesday, January 24, 2023 3:46 PM
> **To:** Engeland, Christine
> **Subject:** CDDIS-22-3819RR Initial Quality Check
>
> Dear Professor Engeland,

In checking in your manuscript submitted to Cell Death & Disease it has come to our attention that the following must be addressed before we can proceed.

1. It has come to our attention that your most recent author list differs from the one in your original submission. We find that the following authors have been added since your initial submission: Johannes Dörner; Hubert Zirngibl; Maximilian Ackermann; Hans M. Kvasnicka.

Please request agreement from all authors including additions and deletions, these can be collected in the following way:

Email your co-authors with the change, and ask them to reply to your email confirming that they agree to these changes. Once you have collected these replies, please combine all of the co-authors' email responses in one document and upload this file to your submission.

Your paper is now available for you to edit, you may access via the following link:

https://mts-cddis.nature.com/cgi-bin/main.plex?el=A2CA3ZRG4C6CMuZ6F1A9ftdTUgP0sjec20MwrNt08A2gZ

(Press/Click on the above link to be automatically sent to the web page.)

Please make the correction(s) as specified above and resubmit your paper following the same steps as before.

If you have any questions please feel free to contact us.

Sincerely,

Editorial Office
Cell Death & Disease
cddisease@springernature.com

This email has been sent through the NPG Manuscript Tracking System NY-610A-NPG&MTS

Confidentiality Statement:

This e-mail is confidential and subject to copyright. Any unauthorised use or disclosure of its contents is prohibited. If you have received this email in error please notify our Manuscript Tracking System Helpdesk team at http://platformsupport.nature.com .

Details of the confidentiality and pre-publicity policy may be found here http://www.nature.com/authors/policies/confidentiality.html

Privacy Policy | Update Profile

Re: Manuscript MV BiKE CDDIS-22-3819: Accepted in Principle

Gmx <gwen.fuchs@gmx.de>

Tue 1/24/2023 9:04 PM

To:Engeland, Christine <christine.engeland@nct-heidelberg.de>;

Dear Christine,

Sure, I confirm. Would it also be possible to change my current Affiliation to the Francis Crick Institute in London as I finished my masters in Stockholm this summer.

How are things going in the lab? Would love to get a little update - and of course congrats to Allesia and you on the paper :)

Best Wishes,
Gwendolin

> Am 24.01.2023 um 17:52 schrieb Engeland, Christine <christine.engeland@nct-heidelberg.de>:

> Dear Colleagues,
>
> Our collaborators from Helios University Clinic Wuppertal Drs.
>
> Johannes Dörner, Hubert Zirngibl, Maximilian Ackermann, Hans M. Kvasnicka
>
> contributed to the experiments performed during the revision of our manuscript.
>
> Please confirm that you agree that they are included as co-authors by responding to this email.
>
> Thank you.
>
> Best regards,
>
> Christine
>
> ---
>
> **From:** cddisease@springernature.com <cddisease@springernature.com>
> **Sent:** Tuesday, January 24, 2023 3:46 PM
> **To:** Engeland, Christine
> **Subject:** CDDIS-22-3819RR Initial Quality Check
>
> Dear Professor Engeland,

In checking in your manuscript submitted to Cell Death & Disease it has come to our attention that the following must be addressed before we can proceed.

1. It has come to our attention that your most recent author list differs from the one in your original submission. We find that the following authors have been added since your initial submission: Johannes Dörner; Hubert Zirngibl; Maximilian Ackermann; Hans M. Kvasnicka.

Please request agreement from all authors including additions and deletions, these can be collected in the following way:

Email your co-authors with the change, and ask them to reply to your email confirming that they agree to these changes. Once you have collected these replies, please combine all of the co-authors' email responses in one document and upload this file to your submission.

Your paper is now available for you to edit, you may access via the following link:

https://mts-cddis.nature.com/cgi-bin/main.plex?el=A2CA3ZRG4C6CMuZ6F1A9ftdTUgP0sjec20MwrNt08A2gZ

(Press/Click on the above link to be automatically sent to the web page.)

Please make the correction(s) as specified above and resubmit your paper following the same steps as before.

If you have any questions please feel free to contact us.

Sincerely,

Editorial Office
Cell Death & Disease
cddisease@springernature.com

This email has been sent through the NPG Manuscript Tracking System NY-610A-NPG&MTS

Confidentiality Statement:

This e-mail is confidential and subject to copyright. Any unauthorised use or disclosure of its contents is prohibited. If you have received this email in error please notify our Manuscript Tracking System Helpdesk team at http://platformsupport.nature.com .

Details of the confidentiality and pre-publicity policy may be found here http://www.nature.com/authors/policies/confidentiality.html

Privacy Policy | Update Profile

AW: Manuscript CDDIS-22-3819R: Accepted in Principle

Dörner, Johannes <Johannes.Doerner@helios-gesundheit.de>

Mi 25.01.2023 12:22

An:Engeland, Christine <Christine.Engeland@uni-wh.de>;

Hallo Christine,

einverstanden. Kannst Du wenn noch möglich das „ö" in meinem Nachnamen in „oe" ändern, so wie hier: Johannes Doerner (0000-0002-0218-4777) (orcid.org) ? Falls nicht ist es nicht dramatisch;-) Nochmal vielen Dank!

Viele Grüße

Johannes

Von: Engeland, Christine <Christine.Engeland@uni-wh.de>
Gesendet: Mittwoch, 25. Januar 2023 11:37
An: Dörner, Johannes <Johannes.Doerner@helios-gesundheit.de>; Zirngibl, Hubert <hubert.zirngibl@helios-gesundheit.de>; Ackermann, Maximilian <Maximilian.Ackermann@helios-gesundheit.de>; Kvasnicka, Hans Michael <HansMichael.Kvasnicka@helios-gesundheit.de>
Betreff: [EXTERN] Manuscript CDDIS-22-3819R: Accepted in Principle

Dear Colleagues,

Please confirm that you agree to be included as authors by responding to this email.

Thank you.

Best regards,
Christine Engeland

From: cddisease@springernature.com <cddisease@springernature.com>
Sent: Tuesday, January 24, 2023 3:46 PM
To: Engeland, Christine
Subject: CDDIS-22-3819RR Initial Quality Check

Dear Professor Engeland,

In checking in your manuscript submitted to Cell Death & Disease it has come to our attention that the following must be addressed before we can proceed.

1. It has come to our attention that your most recent author list differs from the one in your original submission. We find that the following authors have been added since your initial submission: Johannes Dörner; Hubert Zirngibl; Maximilian Ackermann; Hans

M. Kvasnicka.

Please request agreement from all authors including additions and deletions, these can be collected in the following way:

Email your co-authors with the change, and ask them to reply to your email confirming that they agree to these changes. Once you have collected these replies, please combine all of the co-authors’ email responses in one document and upload this file to your submission.

Your paper is now available for you to edit, you may access via the following link:

https://mts-cddis.nature.com/cgi-bin/main.plex?el=A2CA3ZRG4C6CMuZ6F1A9ftdTUgP0sjec20MwrNt08A2gZ

(Press/Click on the above link to be automatically sent to the web page.)

Please make the correction(s) as specified above and resubmit your paper following the same steps as before.

If you have any questions please feel free to contact us.

Sincerely,

Editorial Office
Cell Death & Disease
cddisease@springernature.com

This email has been sent through the NPG Manuscript Tracking System NY-610A-NPG&MTS

Confidentiality Statement:

This e-mail is confidential and subject to copyright. Any unauthorised use or disclosure of its contents is prohibited. If you have received this email in error please notify our Manuscript Tracking System Helpdesk team at http://platformsupport.nature.com .
Details of the confidentiality and pre-publicity policy may be found here http://www.nature.com/authors/policies/confidentiality.html
Privacy Policy | Update Profile

Private Universität Witten/Herdecke gGmbH
Alfred-Herrhausen-Straße 50
D - 58448 Witten

Homepage: www.uni-wh.de

Geschäftsführung: Prof. Dr. Martin Butzlaff (Präsident), Dipl. oec. Jan Peter Nonnenkamp (Kanzler)

Sitz der Gesellschaft: Witten
Handelsregister des Amtsgerichts Bochum Nr. HRB 8671

AW: Manuscript CDDIS-22-3819R: Accepted in Principle

Zirngibl, Hubert <hubert.zirngibl@helios-gesundheit.de>

Mi 25.01.2023 13:14

An:Engeland, Christine <Christine.Engeland@uni-wh.de>;

Dear Ms Engeland
I agree to be included as author by responding to this email.
Best regards,
Hubert Zirngibl

Helios Universitätsklinikum Wuppertal
Universität Witten/Herdecke

Prof. Dr. med. Hubert Zirngibl
Klinik für Allgemein- und Viszeralchirurgie
Senior Consultant

Tel: 0202 896 2474
Fax: 0202 896 2930
Mobil: +49 152 54778213
Mail: hubert.zirngibl@helios-gesundheit.de

Heusnerstr. 40 – 42283 Wuppertal
www.helios-gesundheit.de/wuppertal

Träger: Helios Klinikum Wuppertal GmbH
Geschäftsführer: Dr. Holger Raphael, Prof. Dr. Rudolf Leuwer
Aufsichtsratsvorsitzender: Jörg Reschke
Sitz der Gesellschaft:Wuppertal
Handelsregister: Amtsgericht Wuppertal, HRB 8419

Von: Engeland, Christine <Christine.Engeland@uni-wh.de>
Gesendet: Mittwoch, 25. Januar 2023 11:37
An: Dörner, Johannes <Johannes.Doerner@helios-gesundheit.de>; Zirngibl, Hubert <hubert.zirngibl@helios-gesundheit.de>; Ackermann, Maximilian <Maximilian.Ackermann@helios-gesundheit.de>; Kvasnicka, Hans Michael <HansMichael.Kvasnicka@helios-gesundheit.de>
Betreff: [EXTERN] Manuscript CDDIS-22-3819R: Accepted in Principle

Dear Colleagues,

Please confirm that you agree to be included as authors by responding to this email.

Thank you.

Best regards,
Christine Engeland

From: cddisease@springernature.com <cddisease@springernature.com>
Sent: Tuesday, January 24, 2023 3:46 PM
To: Engeland, Christine
Subject: CDDIS-22-3819RR Initial Quality Check

Dear Professor Engeland,

In checking in your manuscript submitted to Cell Death & Disease it has come to our attention that the following must be addressed before we can proceed.

1. It has come to our attention that your most recent author list differs from the one in your original submission. We find that the following authors have been added since your initial submission: Johannes Dörner; Hubert Zirngibl; Maximilian Ackermann; Hans M. Kvasnicka.

Please request agreement from all authors including additions and deletions, these can be collected in the following way:

Email your co-authors with the change, and ask them to reply to your email confirming that they agree to these changes. Once you have collected these replies, please combine all of the co-authors' email responses in one document and upload this file to your submission.

Your paper is now available for you to edit, you may access via the following link:

https://mts-cddis.nature.com/cgi-bin/main.plex?el=A2CA3ZRG4C6CMuZ6F1A9ftdTUgP0sjec20MwrNt08A2gZ

(Press/Click on the above link to be automatically sent to the web page.)

Please make the correction(s) as specified above and resubmit your paper following the same steps as before.

If you have any questions please feel free to contact us.

Sincerely,

Editorial Office
Cell Death & Disease
cddisease@springernature.com

This email has been sent through the NPG Manuscript Tracking System NY-610A-NPG&MTS

Confidentiality Statement:

This e-mail is confidential and subject to copyright. Any unauthorised use or disclosure of its contents is prohibited. If you have received this email in error please notify our Manuscript Tracking System Helpdesk team at http://platformsupport.nature.com . Details of the confidentiality and pre-publicity policy may be found here http://www.nature.com/authors/policies/confidentiality.html
Privacy Policy | Update Profile

Private Universität Witten/Herdecke gGmbH
Alfred-Herrhausen-Straße 50
D - 58448 Witten

Homepage: www.uni-wh.de

Geschäftsführung: Prof. Dr. Martin Butzlaff (Präsident), Dipl. oec. Jan Peter Nonnenkamp (Kanzler)

Sitz der Gesellschaft: Witten
Handelsregister des Amtsgerichts Bochum Nr. HRB 8671

AW: Manuscript CDDIS-22-3819R: Accepted in Principle

Ackermann, Maximilian <Maximilian.Ackermann@helios-gesundheit.de>

Mi 25.01.2023 12:16

An:Engeland, Christine <Christine.Engeland@uni-wh.de>;

Dear Professor Engeland,

thank you very much for your mail. I, hereby, confirm my authorship in the attached publication in Cell Death & Disease.

Thank you very much and kind regards,

Maximilian Ackermann

Von: Engeland, Christine <Christine.Engeland@uni-wh.de>
Gesendet: Mittwoch, 25. Januar 2023 11:37
An: Dörner, Johannes <Johannes.Doerner@helios-gesundheit.de>; Zirngibl, Hubert <hubert.zirngibl@helios-gesundheit.de>; Ackermann, Maximilian <Maximilian.Ackermann@helios-gesundheit.de>; Kvasnicka, Hans Michael <HansMichael.Kvasnicka@helios-gesundheit.de>
Betreff: [EXTERN] Manuscript CDDIS-22-3819R: Accepted in Principle

Dear Colleagues,

Please confirm that you agree to be included as authors by responding to this email.

Thank you.

Best regards,
Christine Engeland

From: cddisease@springernature.com <cddisease@springernature.com>
Sent: Tuesday, January 24, 2023 3:46 PM
To: Engeland, Christine
Subject: CDDIS-22-3819RR Initial Quality Check

Dear Professor Engeland,

In checking in your manuscript submitted to Cell Death & Disease it has come to our attention that the following must be addressed before we can proceed.

1. It has come to our attention that your most recent author list differs from the one in your original submission. We find that the following authors have been added since your initial submission: Johannes Dörner; Hubert Zirngibl; Maximilian Ackermann; Hans M. Kvasnicka.

Please request agreement from all authors including additions and deletions, these

can be collected in the following way:

Email your co-authors with the change, and ask them to reply to your email confirming that they agree to these changes. Once you have collected these replies, please combine all of the co-authors' email responses in one document and upload this file to your submission.

Your paper is now available for you to edit, you may access via the following link:

https://mts-cddis.nature.com/cgi-bin/main.plex?el=A2CA3ZRG4C6CMuZ6F1A9ftdTUgP0sjec20MwrNt08A2gZ

(Press/Click on the above link to be automatically sent to the web page.)

Please make the correction(s) as specified above and resubmit your paper following the same steps as before.

If you have any questions please feel free to contact us.

Sincerely,

Editorial Office
Cell Death & Disease
cddisease@springernature.com

This email has been sent through the NPG Manuscript Tracking System NY-610A-NPG&MTS

Confidentiality Statement:

This e-mail is confidential and subject to copyright. Any unauthorised use or disclosure of its contents is prohibited. If you have received this email in error please notify our Manuscript Tracking System Helpdesk team at http://platformsupport.nature.com .
Details of the confidentiality and pre-publicity policy may be found here http://www.nature.com/authors/policies/confidentiality.html
Privacy Policy | Update Profile

Private Universität Witten/Herdecke gGmbH
Alfred-Herrhausen-Straße 50
D - 58448 Witten

Homepage: www.uni-wh.de

Geschäftsführung: Prof. Dr. Martin Butzlaff (Präsident), Dipl. oec. Jan Peter Nonnenkamp (Kanzler)

Sitz der Gesellschaft: Witten

Handelsregister des Amtsgerichts Bochum Nr. HRB 8671

AW: Manuscript CDDIS-22-3819R: Accepted in Principle

Kvasnicka, Hans Michael <HansMichael.Kvasnicka@helios-gesundheit.de>

Mi 25.01.2023 20:00

An:Engeland, Christine <Christine.Engeland@uni-wh.de>; Dörner, Johannes <Johannes.Doerner@helios-gesundheit.de>; Zirngibl, Hubert <hubert.zirngibl@helios-gesundheit.de>; Ackermann, Maximilian <Maximilian.Ackermann@helios-gesundheit.de>;

Dear Professor Engeland,

it is my honor and pleasure to be included as author to this important paper.

Best regards

HM Kvasnicka

____

Helios Universitätsklinikum Wuppertal
Universität Witten/Herdecke

Prof. Dr. med. Hans Michael Kvasnicka
Direktor Institut für Pathologie und Molekularpathologie

Tel. : +49 (202) 896 2849
Fax : +49 (202) 896 2739

Heusnerstr. 40 - 42283 Wuppertal
www.helios-gesundheit.de/wuppertal
Träger: Helios Klinikum Wuppertal GmbH
Geschäftsführer: Dr. Holger Raphael, Prof. Dr. med. Rudolf Leuwer
Aufsichtsratsvorsitzender: Jörg Reschke
Sitz der Gesellschaft: Wuppertal
Handelsregister: Amtsgericht Wuppertal, HRB 841

Von: Engeland, Christine <Christine.Engeland@uni-wh.de>
Gesendet: Mittwoch, 25. Januar 2023 11:37
An: Dörner, Johannes <Johannes.Doerner@helios-gesundheit.de>; Zirngibl, Hubert <hubert.zirngibl@helios-gesundheit.de>; Ackermann, Maximilian <Maximilian.Ackermann@helios-gesundheit.de>; Kvasnicka, Hans Michael <HansMichael.Kvasnicka@helios-gesundheit.de>
Betreff: [EXTERN] Manuscript CDDIS-22-3819R: Accepted in Principle

Dear Colleagues,

Please confirm that you agree to be included as authors by responding to this email.

Thank you.

Best regards,

Christine Engeland

From: cddisease@springernature.com <cddisease@springernature.com>
Sent: Tuesday, January 24, 2023 3:46 PM
To: Engeland, Christine
Subject: CDDIS-22-3819RR Initial Quality Check

Dear Professor Engeland,

In checking in your manuscript submitted to Cell Death & Disease it has come to our attention that the following must be addressed before we can proceed.

1. It has come to our attention that your most recent author list differs from the one in your original submission. We find that the following authors have been added since your initial submission: Johannes Dörner; Hubert Zirngibl; Maximilian Ackermann; Hans M. Kvasnicka.

Please request agreement from all authors including additions and deletions, these can be collected in the following way:

Email your co-authors with the change, and ask them to reply to your email confirming that they agree to these changes. Once you have collected these replies, please combine all of the co-authors' email responses in one document and upload this file to your submission.

Your paper is now available for you to edit, you may access via the following link:

https://mts-cddis.nature.com/cgi-bin/main.plex?el=A2CA3ZRG4C6CMuZ6F1A9ftdTUgP0sjec20MwrNt08A2gZ

(Press/Click on the above link to be automatically sent to the web page.)

Please make the correction(s) as specified above and resubmit your paper following the same steps as before.

If you have any questions please feel free to contact us.

Sincerely,

Editorial Office
Cell Death & Disease
cddisease@springernature.com

This email has been sent through the NPG Manuscript Tracking System NY-610A-NPG&MTS

Confidentiality Statement:

This e-mail is confidential and subject to copyright. Any unauthorised use or disclosure of its contents is prohibited. If you have received this email in error please notify our Manuscript Tracking System Helpdesk team at http://platformsupport.nature.com . Details of the confidentiality and pre-publicity policy may be found here http://www.nature.com/authors/policies/confidentiality.html
Privacy Policy | Update Profile

Private Universität Witten/Herdecke gGmbH
Alfred-Herrhausen-Straße 50
D - 58448 Witten

Homepage: www.uni-wh.de

Geschäftsführung: Prof. Dr. Martin Butzlaff (Präsident), Dipl. oec. Jan Peter Nonnenkamp (Kanzler)

Sitz der Gesellschaft: Witten
Handelsregister des Amtsgerichts Bochum Nr. HRB 8671

Re: Manuscript MV BiKE CDDIS-22-3819: Accepted in Principle

Chester, Kerry <k.chester@ucl.ac.uk>

Tue 1/24/2023 7:38 PM

To:Engeland, Christine <christine.engeland@nct-heidelberg.de>;

Cc:Schäfer, Theresa <theresa.schaefer@dkfz-heidelberg.de>; gwen.fuchs@gmx.de <gwen.fuchs@gmx.de>; Jäger, Dirk <dirk.jaeger@nct-heidelberg.de>; Ball, Claudia <claudia.ball@nct-dresden.de>; Ungerechts, Guy <guy.ungerechts@nct-heidelberg.de>; Flörchinger, Alessia <a.floerchinger@dkfz-heidelberg.de>; Jessica Klein <jessica.e.klein@web.de>; Finkbeiner, Maximiliane <Maximiliane.Finkbeiner@uni-wh.de>;

I confirm

Best wishes

Kerry Chester

> On 24 Jan 2023, at 17:52, Engeland, Christine <christine.engeland@nct-heidelberg.de> wrote:

> ⚠ Caution: External sender
>
> Dear Colleagues,
>
> Our collaborators from Helios University Clinic Wuppertal Drs.
>
> Johannes Dörner, Hubert Zirngibl, Maximilian Ackermann, Hans M. Kvasnicka
>
> contributed to the experiments performed during the revision of our manuscript.
>
> Please confirm that you agree that they are included as co-authors by responding to this email.
>
> Thank you.
>
> Best regards,
>
> Christine
>
> ---
>
> **From:** cddisease@springernature.com <cddisease@springernature.com>
> **Sent:** Tuesday, January 24, 2023 3:46 PM

To: Engeland, Christine
Subject: CDDIS-22-3819RR Initial Quality Check

Dear Professor Engeland,

In checking in your manuscript submitted to Cell Death & Disease it has come to our attention that the following must be addressed before we can proceed.

1. It has come to our attention that your most recent author list differs from the one in your original submission. We find that the following authors have been added since your initial submission: Johannes Dörner; Hubert Zirngibl; Maximilian Ackermann; Hans M. Kvasnicka.

Please request agreement from all authors including additions and deletions, these can be collected in the following way:

Email your co-authors with the change, and ask them to reply to your email confirming that they agree to these changes. Once you have collected these replies, please combine all of the co-authors' email responses in one document and upload this file to your submission.

Your paper is now available for you to edit, you may access via the following link:

https://mts-cddis.nature.com/cgi-bin/main.plex?el=A2CA3ZRG4C6CMuZ6F1A9ftdTUgP0sjec20MwrNt08A2gZ

(Press/Click on the above link to be automatically sent to the web page.)

Please make the correction(s) as specified above and resubmit your paper following the same steps as before.

If you have any questions please feel free to contact us.

Sincerely,

Editorial Office
Cell Death & Disease
cddisease@springernature.com

This email has been sent through the NPG Manuscript Tracking System NY-610A-NPG&MTS

Confidentiality Statement:

This e-mail is confidential and subject to copyright. Any unauthorised use or disclosure of its contents is prohibited. If you have received this email in error please notify our Manuscript Tracking System Helpdesk team at http://platformsupport.nature.com .

Details of the confidentiality and pre-publicity policy may be found here http://www.nature.com/authors/policies/confidentiality.html

Privacy Policy | Update Profile

Re: Manuscript MV BiKE CDDIS-22-3819: Accepted in Principle

Jäger, Dirk

Tue 1/24/2023 7:17 PM

To:Engeland, Christine <christine.engeland@nct-heidelberg.de>;

Fine with me!
Best,
DJ

Prof. Dr. med. Dirk Jäger

Director Medical Oncology
University Medical Center Heidelberg

Head of DKFZ Clinical Cooperation Unit „Applied Tumor-Immunity“ (D120)

National Center for Tumor Diseases (NCT)
University Medical Center Heidelberg
Im Neuenheimer Feld 460
69120 Heidelberg
Germany

Phone: + 49-6221-567228
Fax: + 49-6221-567225

dirk.jaeger@nct-heidelberg.de
www.nct-heidelberg.de

NCT
NATIONALES CENTRUM FÜR TUMORERKRANKUNGEN HEIDELBERG
getragen von:
Deutsches Krebsforschungszentrum
Universitätsklinikum Heidelberg
Medizinische Fakultät
Deutsche Krebshilfe

Von: "Engeland, Christine" <christine.engeland@nct-heidelberg.de>
Datum: Dienstag, 24. Januar 2023 um 18:52
An: "Schäfer, Theresa" <theresa.schaefer@dkfz-heidelberg.de>, "gwen.fuchs@gmx.de" <gwen.fuchs@gmx.de>, "k.chester@ucl.ac.uk" <k.chester@ucl.ac.uk>, "Jäger, Dirk" <dirk.jaeger@nct-heidelberg.de>, "Ball, Claudia" <claudia.ball@nct-dresden.de>, "Ungerechts, Guy" <guy.ungerechts@nct-heidelberg.de>
Cc: "Flörchinger, Alessia" <a.floerchinger@dkfz-heidelberg.de>, Jessica Klein <jessica.e.klein@web.de>, "Finkbeiner, Maximiliane" <Maximiliane.Finkbeiner@uni-wh.de>
Betreff: Re: Manuscript MV BiKE CDDIS-22-3819: Accepted in Principle

Dear Colleagues,

Our collaborators from Helios University Clinic Wuppertal Drs.

Johannes Dörner, Hubert Zirngibl, Maximilian Ackermann, Hans M. Kvasnicka

contributed to the experiments performed during the revision of our manuscript.

Please confirm that you agree that they are included as co-authors by responding to this email.

Thank you.

Best regards,

Christine

From: cddisease@springernature.com <cddisease@springernature.com>
Sent: Tuesday, January 24, 2023 3:46 PM
To: Engeland, Christine
Subject: CDDIS-22-3819RR Initial Quality Check

Dear Professor Engeland,

In checking in your manuscript submitted to Cell Death & Disease it has come to our attention that the following must be addressed before we can proceed.

1. It has come to our attention that your most recent author list differs from the one in your original submission. We find that the following authors have been added since your initial submission: Johannes Dörner; Hubert Zirngibl; Maximilian Ackermann; Hans M. Kvasnicka.

Please request agreement from all authors including additions and deletions, these can be collected in the following way:

Email your co-authors with the change, and ask them to reply to your email confirming that they agree to these changes. Once you have collected these replies, please combine all of the co-authors' email responses in one document and upload this file to your submission.

Your paper is now available for you to edit, you may access via the following link:

https://mts-cddis.nature.com/cgi-bin/main.plex?el=A2CA3ZRG4C6CMuZ6F1A9ftdTUgP0sjec20MwrNt08A2gZ

(Press/Click on the above link to be automatically sent to the web page.)

Please make the correction(s) as specified above and resubmit your paper following the same steps as before.

If you have any questions please feel free to contact us.

Sincerely,

Editorial Office
Cell Death & Disease
cddisease@springernature.com

This email has been sent through the NPG Manuscript Tracking System NY-610A-NPG&MTS

Confidentiality Statement:

This e-mail is confidential and subject to copyright. Any unauthorised use or disclosure of its contents is prohibited. If you have received this email in error please notify our Manuscript Tracking System Helpdesk team at http://platformsupport.nature.com .

Details of the confidentiality and pre-publicity policy may be found here http://www.nature.com/authors/policies/confidentiality.html

Privacy Policy | Update Profile

Re: Manuscript MV BiKE CDDIS-22-3819: Accepted in Principle

Ball, Claudia

Tue 1/24/2023 7:25 PM

To:Engeland, Christine <christine.engeland@nct-heidelberg.de>;

Dear Christine, I agree.
Best, Claudia

Am 24.01.2023 18:52 schrieb "Engeland, Christine" <christine.engeland@nct-heidelberg.de>:
Dear Colleagues,

Our collaborators from Helios University Clinic Wuppertal Drs.
Johannes Dörner, Hubert Zirngibl, Maximilian Ackermann, Hans M. Kvasnicka
contributed to the experiments performed during the revision of our manuscript.

Please confirm that you agree that they are included as co-authors by responding to this email.

Thank you.
Best regards,
Christine

From: cddisease@springernature.com <cddisease@springernature.com>
Sent: Tuesday, January 24, 2023 3:46 PM
To: Engeland, Christine
Subject: CDDIS-22-3819RR Initial Quality Check

Dear Professor Engeland,

In checking in your manuscript submitted to Cell Death & Disease it has come to our attention that the following must be addressed before we can proceed.

1. It has come to our attention that your most recent author list differs from the one in your original submission. We find that the following authors have been added since your initial submission: Johannes Dörner; Hubert Zirngibl; Maximilian Ackermann; Hans M. Kvasnicka.

Please request agreement from all authors including additions and deletions, these can be collected in the following way:

Email your co-authors with the change, and ask them to reply to your email confirming that they agree to these changes. Once you have collected these replies, please combine all of the co-authors' email responses in one document and upload this file to your submission.

Your paper is now available for you to edit, you may access via the following link:

https://mts-cddis.nature.com/cgi-bin/main.plex?el=A2CA3ZRG4C6CMuZ6F1A9ftdTUgP0sjec20MwrNt08A2gZ

(Press/Click on the above link to be automatically sent to the web page.)

Please make the correction(s) as specified above and resubmit your paper following the same steps as before.

If you have any questions please feel free to contact us.

Sincerely,

Editorial Office
Cell Death & Disease
cddisease@springernature.com

This email has been sent through the NPG Manuscript Tracking System NY-610A-NPG&MTS

Confidentiality Statement:

This e-mail is confidential and subject to copyright. Any unauthorised use or disclosure of its contents is prohibited. If you have received this email in error please notify our Manuscript Tracking System Helpdesk team at http://platformsupport.nature.com .
Details of the confidentiality and pre-publicity policy may be found here http://www.nature.com /authors/policies/confidentiality.html
Privacy Policy | Update Profile

Re: Manuscript MV BiKE CDDIS-22-3819: Accepted in Principle

Ungerechts, Guy

Tue 1/24/2023 8:49 PM

To:Engeland, Christine <christine.engeland@nct-heidelberg.de>;

Cc:Schäfer, Theresa <theresa.schaefer@dkfz-heidelberg.de>; gwen.fuchs@gmx.de <gwen.fuchs@gmx.de>; k.chester@ucl.ac.uk <k.chester@ucl.ac.uk>; Jäger, Dirk <dirk.jaeger@nct-heidelberg.de>; Ball, Claudia <claudia.ball@nct-dresden.de>; Flörchinger, Alessia <a.floerchinger@dkfz-heidelberg.de>; Jessica Klein <jessica.e.klein@web.de>; Finkbeiner, Maximiliane <Maximiliane.Finkbeiner@uni-wh.de>;

Confirmed.

Best,
Guy

Prof. Dr. med. Dr. rer. nat. Guy Ungerechts
Stellv. Ärztlicher Direktor
Leitender Oberarzt
Abteilung Medizinische Onkologie
Nationales Centrum für Tumorerkrankungen (NCT) Heidelberg
Universitätsklinikum Heidelberg
Im Neuenheimer Feld 460
D-69120 Heidelberg
Germany

Tel.: +49 6221 567229
Fax: +49 6221 565218
Email: guy.ungerechts@nct-heidelberg.de

> Am 24.01.2023 um 18:52 schrieb Engeland, Christine <christine.engeland@nct-heidelberg.de>:

> Dear Colleagues,

Our collaborators from Helios University Clinic Wuppertal Drs.

Johannes Dörner, Hubert Zirngibl, Maximilian Ackermann, Hans M. Kvasnicka

contributed to the experiments performed during the revision of our manuscript.

Please confirm that you agree that they are included as co-authors by responding to this email.

Thank you.

Best regards,

Christine

From: cddisease@springernature.com <cddisease@springernature.com>
Sent: Tuesday, January 24, 2023 3:46 PM
To: Engeland, Christine
Subject: CDDIS-22-3819RR Initial Quality Check

Dear Professor Engeland,

In checking in your manuscript submitted to Cell Death & Disease it has come to our attention that the following must be addressed before we can proceed.

1. It has come to our attention that your most recent author list differs from the one in your original submission. We find that the following authors have been added since your initial submission: Johannes Dörner; Hubert Zirngibl; Maximilian Ackermann; Hans M. Kvasnicka.

Please request agreement from all authors including additions and deletions, these can be collected in the following way:

Email your co-authors with the change, and ask them to reply to your email confirming that they agree to these changes. Once you have collected these replies, please combine all of the co-authors' email responses in one document and upload this file to your submission.

Your paper is now available for you to edit, you may access via the following link:

https://mts-cddis.nature.com/cgi-bin/main.plex?el=A2CA3ZRG4C6CMuZ6F1A9ftdTUgP0sjec20MwrNt08A2gZ

(Press/Click on the above link to be automatically sent to the web page.)

Please make the correction(s) as specified above and resubmit your paper following the same steps as before.

If you have any questions please feel free to contact us.

Sincerely,

Editorial Office
Cell Death & Disease
cddisease@springernature.com

This email has been sent through the NPG Manuscript Tracking System NY-610A-NPG&MTS

Confidentiality Statement:

This e-mail is confidential and subject to copyright. Any unauthorised use or disclosure of its contents is prohibited. If you have received this email in error please notify our Manuscript Tracking System Helpdesk team at http://platformsupport.nature.com .

Details of the confidentiality and pre-publicity policy may be found here http://www.nature.com/authors/policies/confidentiality.html

Privacy Policy | Update Profile
